# Shoreline wave breaking strongly enhances the coastal sea spray aerosol population: Climate and air quality implications

**DOI:** 10.1126/sciadv.adw0343

**Published:** 2025-08-27

**Authors:** Shengqian Zhou, Matthew Salter, Timothy Bertram, Eduardo Brito Azevedo, Francisco Reis, Jian Wang

**Affiliations:** ^1^ Center for Aerosol Science and Engineering, Department of Energy, Environmental and Chemical Engineering, Washington University in St. Louis, St. Louis, MO, USA.; ^2^ Department of Environmental Science, Stockholm University, Stockholm, Sweden.; ^3^ Baltic Sea Centre, Stockholm University, Stockholm, Sweden.; ^4^ Department of Chemistry, University of Wisconsin, Madison, WI, USA.; ^5^ Institute of Agricultural and Environmental Research and Technology (IITAA), University of the Azores, Angra do Heroísmo, Azores, Portugal.; ^6^ Environmental Observatory of the Azores, Angra do Heroísmo, Azores, Portugal.

## Abstract

Sea spray aerosol (SSA) emission is a major source of atmospheric aerosols, influencing global climate and coastal air quality. Much of our knowledge about SSA derives from coastal observations near shorelines, but whether and when these observations accurately represent open oceans remain unclear. Here, we show that strong nearshore SSA production during high-wave periods greatly enhances downwind cloud condensation nuclei (CCN) and aerosol mass concentrations. Strong shoreline wave breaking is widespread globally, and swell waves, which are decoupled from local winds, play a dominant role in many coastal regions. Therefore, extrapolating results based on coastal measurements to open oceans may significantly overestimate SSA concentration and its contribution to CCN and, by extension, the impact of SSA on clouds and climate. Additionally, the strong enhancement of coastal aerosol population by shoreline wave breaking and its environmental impact on coastal communities cannot be captured by current regional models, which do not parameterize nearshore SSA generation using wave energy or completely neglect it.

## Teaser

Wave energy, not wind speed, controls sea spray production near shorelines, with implications for coastal aerosol populations.

## INTRODUCTION

Atmospheric aerosols substantially affect both the climate system and air quality (*1*, *2*). Sea spray aerosol (SSA), emitted from ocean wave breaking and associated bubble bursting (*3*, *4*), is one of the largest global aerosol sources by mass (*5*) and a critical component that couples the ocean and atmosphere in the Earth system (*6*–*8*). It is well recognized that SSA dominates the aerosol mass and scattering over remote oceans, leading to a notable direct radiative effect (*9*). However, the indirect radiative effects of SSA are less understood due to substantial uncertainties in SSA’s contribution to the number concentration of cloud-forming particles, i.e., cloud condensation nuclei (CCN), in the marine boundary layer (MBL) (*10*). Prior observational studies examining the contribution of SSA to CCN have drawn divergent conclusions. Studies using lognormal fitting to isolate SSA from size distributions over open oceans suggest relatively minor contributions; for instance, Quinn *et al.* (*11*) found that SSA contributed less than 30% of total CCN across most global oceans, and similar results were found in Eastern Pacific off the coast of California (*12*). Contrarily, other studies suggest that the lognormal fitting alone may substantially underestimate SSA number concentration (*13*). Size-resolved hygroscopicity measurements at the coastal sites near shorelines indicate that SSA can dominate sub-100-nm particles and CCN populations (*13*–*15*). However, whether these results based on coastal observations apply to SSA over open oceans remains unclear.

Ocean waves are primarily generated by wind blowing across the ocean surface, transferring energy from the atmosphere into the ocean, and the characteristics and spatiotemporal dynamics of these freshly generated waves (i.e., wind waves) are coupled with local wind fields when they are in equilibrium (*16*, *17*). In open ocean regions, SSA predominantly originates from the breaking of wind waves under strong wind conditions, making SSA emission flux highly dependent on local wind speed (*4*). When winds subside or as wind waves travel out of the generation area, these waves transform into long-period, smoother swell waves that are decoupled from local winds (*18*, *19*). Whereas the presence of swell waves can influence wind wave breaking by disturbing surface roughness and altering interactions between the wave field and the overlying air flow (*20*, *21*), swell waves typically do not break in deep open oceans themselves. On the other hand, swell waves can travel long distances across the ocean basin (*22*, *23*), and upon approaching shorelines, they can break in shallow waters (i.e., the surf zone) due to interactions with the ocean floor (*18*, *24*). Swell waves can also break when directly hitting steep rocky shorelines (*25*). Additionally, the breaking of wind waves is also greatly enhanced at shorelines through these processes. Moreover, nearshore regions generally exhibit greater air entrainment and bubble generation per breaking event, largely due to a higher prevalence of energetic plunging breakers, which produce more air bubbles than wind-driven spilling breakers in open oceans (*26*, *27*). As a result, the shoreline wave breaking generates substantially higher SSA emission fluxes, strongly elevating downwind aerosol concentrations (*28*–*30*).

Previous studies on the impact of shoreline wave breaking have mainly focused on aerosol mass concentrations (*31*, *32*), scattering properties (*33*, *34*), or the number concentrations of particles larger than 150 nm (*28*, *29*, *35*, *36*). There is now a lack of systematic investigations into the influence on the CCN population, which has a large contribution from sub-100-nm particles. Furthermore, existing studies are mostly limited to short-term observations lasting only a few days, which do not allow for comprehensive evaluations under the full range of representative conditions. Given that many SSA-related studies rely on coastal observations and those observations have been used to investigate the dependence of SSA concentration or flux on environmental variables (*13*, *37*–*39*), evaluate the SSA concentrations simulated by global models with different SSA source functions (*40*–*42*), and assess the climate effects of SSA (*10*, *13*), it is imperative to understand how shoreline wave breaking affects those observations and whether and when they accurately represent open ocean conditions.

Beyond global climate effects, SSA is also an important constituent of the aerosols in coastal regions, influencing coastal air quality and meteorology (*43*, *44*). SSA production can introduce chemical and biological pollutants from seawater into the atmosphere, affecting human health (*45*–*48*). These pollutants are often more abundant in coastal waters, and many coastal areas have high population density near shorelines. Consequently, although the overall global flux of SSA from shoreline wave breaking (referred to as nearshore SSA) might be smaller than that from open oceans, its health impact can be disproportionately strong. However, previous modeling studies on coastal air quality have rarely accounted for this process. A better understanding of nearshore SSA generation is crucial for assessing and predicting its potential environmental impact on coastal communities.

In this study, we examine coastal aerosol properties using multiyear measurements. We present clear evidence that nearshore SSA generation strongly enhances both CCN population (dominated by sub-200-nm particles) and aerosol mass concentration (dominated by coarse particles), with these enhancements controlled primarily by wave height (i.e., a surrogate of wave energy) rather than wind speed. Analysis of global wave statistics indicates that such wave-driven SSA production is common at coastlines. We demonstrate that nearshore SSA production, which has been largely overlooked, must be considered both when using coastal measurements to understand open ocean SSA properties and when predicting coastal aerosol populations and their environmental impacts on coastal communities.

## RESULTS

### SSA from shoreline wave breaking

**Fig. 1.**
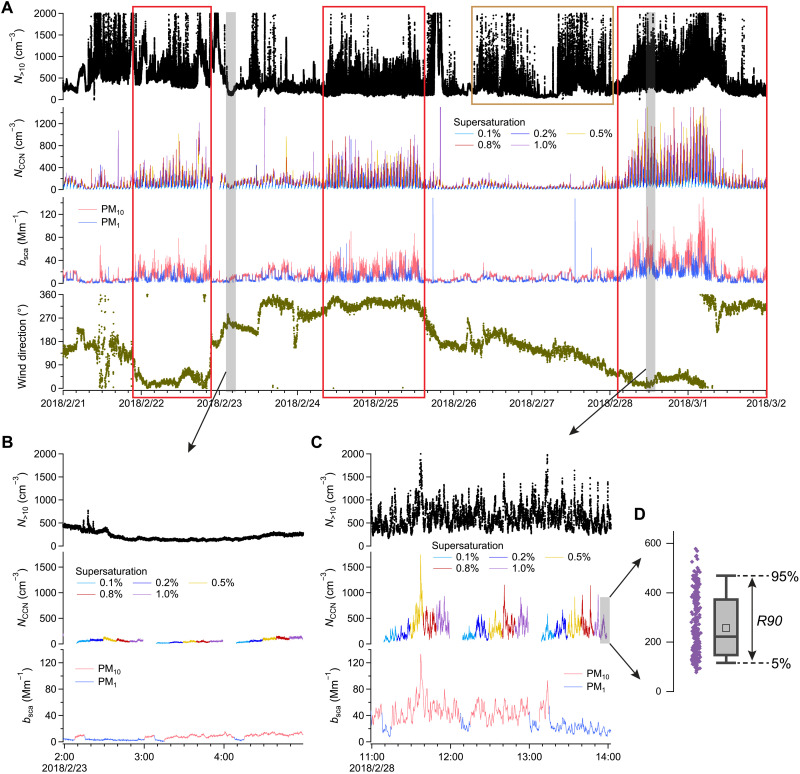
Examples of abnormally strong fluctuations in aerosol concentrations at the ENA site. (**A**) Time series of total number concentration of particles with diameters larger than 10 nm (*N*_>10_), CCN concentration (*N*_CCN_), scattering coefficient (*b*_sca_) at 550 nm, and wind direction from 21 February to 2 March 2018. Red boxes highlight the periods with abnormally strong fluctuations in aerosol concentrations, and the brown box indicates an example period influenced by local pollution. (**B** and **C**) Zoomed-in periods showing examples of background aerosol fluctuation in (B) and abnormally strong fluctuation in (C), respectively. (**D**) The definition of *R90*, a metric of CCN fluctuation intensity.

In remote marine environments, aerosol concentrations typically do not vary strongly in short periods due to the absence of localized sources. However, strong fluctuations of aerosol concentration are frequently observed at the eastern North Atlantic (ENA) site, a long-term atmospheric observatory on Graciosa Island in the remote ENA (Materials and Methods and fig. S1). The Graciosa Island is a volcanic island with rocky shoreline, and the ENA site is located 470 m to the northern shoreline. The examples illustrating the contrast between such strong-fluctuation events and background periods are shown in Fig. 1. During these events, the total aerosol number concentration above 10 nm (*N*_>10_) and CCN concentration (*N*_CCN_) show rapid variations up to 1000 cm^−3^ over timescales of 1 to 5 min. Aerosol scattering coefficients (*b*_sca_) simultaneously exhibit strong fluctuations of up to 100 Mm^−1^ over similar timescales. Some of these events can last more than 24 hours. The above phenomena indicate a strong local aerosol source. We exclude local anthropogenic pollution because the winds are primarily from the north without passing through populated areas or the port during these events. Moreover, the fresh primary aerosols from local pollution are mostly ultrafine particles enriched with less-hygroscopic organics and black carbon, which do not act as CCN under typical supersaturations (*49*). Thus, while local pollution may greatly enhance both *N*_>10_ and its fluctuation, it has little impact on *N*_CCN_ or scattering (Fig. 1 and Supplementary Text). Emissions from the international shipping corridor located north of Graciosa Island are also unlikely the cause for these strong-fluctuation events. This is because plumes from the shipping corridor are highly diluted and mixed during their transport, typically over tens of kilometers, before reaching the ENA site. Furthermore, with average cruising speeds, ships can travel hundreds of kilometers in 24 hours, making it highly improbable to continuously affect the aerosol measurements over extended periods lasting more than 1 day.

**Fig. 2.**
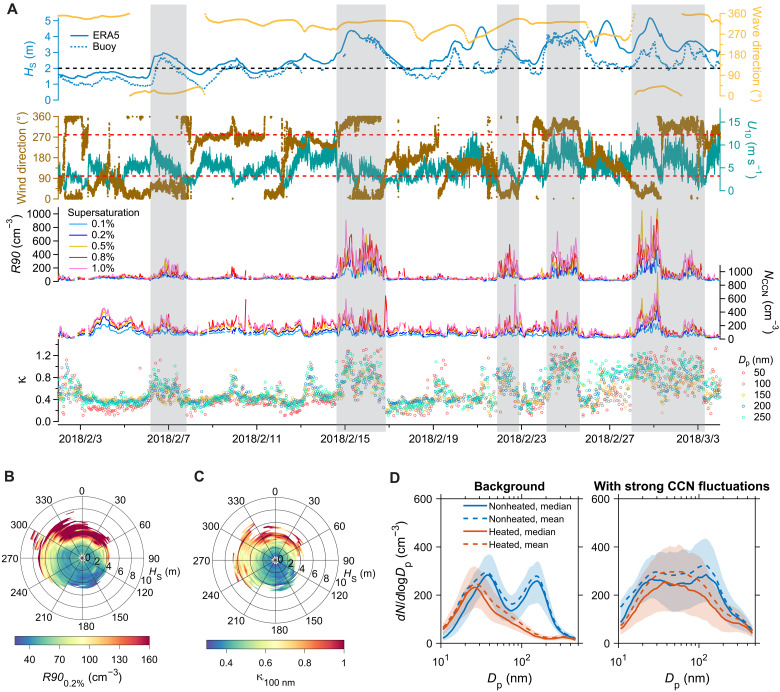
Identification of the SSA generated by shoreline wave breaking at the ENA site. (**A**) Time series of significant wave height (*H*_S_), wave direction, 10-m wind direction, 10-m wind speed (*U*_10_), CCN fluctuation intensity (*R90*), CCN concentration (*N*_CCN_), and aerosol hygroscopicity parameter (κ) from 2 February to 3 March 2018. The black dashed line represents *H*_S_ = 2.0 m. Both *H*_S_ from ECMWF Reanalysis v5 (ERA5) and buoy observations are shown. Please see Materials and Methods and fig. S2 for the comparison between *H*_S_ from ERA5 and buoy observations. The wind direction below lower red dash line (100°) and above upper red dash line (280°) indicates typical onshore wind under which air mass travels less than 1.8 km from the shoreline to the site. The gray shaded regions represent the time periods with abnormally strong CCN fluctuations, which indicate strong impact of nearshore SSA. (**B**) Bivariate polar plot showing the dependence of *R90* (supersaturation, 0.2%) on wind direction and *H*_S_ from 2013 to 2023. (**C**) The same as (B) but for κ values of particles with dry diameter of 100 nm. The *H*_S_ data for [(B) and (C)] are from ERA5. (**D**) Distinct number size distributions of ambient aerosol samples (blue) and those heated to 300°C (red) during background periods and the periods with strong CCN fluctuations, respectively. Here, *D*_p_ represents the particle dry diameter. The solid and dash lines represent the median and mean size distributions, respectively, while the shaded regions represent the ranges between the 25th and 75th percentiles. The data in plots are from 2 February to 3 March 2018, but the periods affected by local anthropogenic pollutions and new particle formations are excluded.

We attribute these events to nearshore SSA on the basis of the following evidence. Figure 2A shows five long-lasting (>12 hours) events with strong CCN fluctuations observed from 2 February to 3 March 2018 (additional examples in fig. S3). Here, the CCN fluctuation is quantified by the difference between the 95th and 5th percentiles of CCN concentration at a fixed supersaturation within a 5-min time window (referred to as *R90*, see details in Materials and Methods). All these events coincide with periods of strong ocean waves from the north and onshore wind, specifically, when the significant wave height (*H*_S_, corresponding to total waves unless stated otherwise) exceeds 2 m and the wind is from the north shore (wind direction of >280° or <100°). During these events, the hygroscopicity parameter (κ) of fine-mode particles (diameter from 50 to 250 nm) markedly increases, often surpassing 1, and the nonvolatile aerosol number fraction becomes elevated compared to background periods without strong aerosol fluctuations, indicating a substantial increase of the SSA fraction. Statistical analyses over a 10-year period (2013–2023) clearly show strong CCN fluctuations and elevated κ values under high-wave and onshore-wind conditions (Fig. 2, B and C). The occurrence of these events is not directly linked to the 10-m surface wind speed (*U*_10_), a key driver of SSA emission in open oceans (*4*). For example, the highest *R90* among these five events takes place at the early morning of 1 March when *U*_10_ is decreasing to below 5 m s^−1^. Average values within two-dimensional bins of *H*_S_ and *U*_10_ demonstrate that both CCN fluctuation and κ strongly increase with increasing *H*_S_ at any given *U*_10_ level, even below 3 m s^−1^, while positive correlations with *U*_10_ are absent at nearly all *H*_S_ levels (fig. S4, B and D). Similar results emerge when replacing *U*_10_ with wind-wave Reynolds number ( ${\text{Re}}_{H_{w}}$ ), which incorporates the effects of sea state and has been suggested as a better determinant of open-ocean whitecap coverage and SSA emission (Supplementary Text and fig. S5) (*39*). These results indicate that open-ocean SSA is not the primary cause of the concentration fluctuations. Furthermore, if these events were due to open-ocean SSA emission, then there would be no strong dependence on wind direction, as open-ocean SSAs are generated over a large area (*4*) and the travel time of an air mass over the island (<11 km) before it reaches the site is much shorter than the atmospheric lifetime of CCN-size particles for all wind directions. Therefore, these events exhibiting strong fluctuations and elevated SSA fraction are attributed to SSA produced from shoreline wave breaking.

Under high-wave conditions (i.e., *H*_S_ > 2 m), intensified wave breaking near the shoreline generates a large SSA flux (*24*, *29*–*31*). In this region, the periods of individual wave phases typically range from 4.9 to 9.5 s (5th to 95th percentiles of the 10-year local buoy measurement data), and the wave amplitudes can vary substantially within wave groups over periods of 1 to 5 min, resulting in varying shoreline wave breaking intensity and subsequent SSA emission flux on timescales ranging from seconds to minutes. With a wind speed of 5 m s^−1^, it takes ~1.5 to 6 min for air parcels to travel from the northern shoreline to the ENA site, depending on wind direction. During these short periods, air parcels carrying nearshore SSA with varying concentrations do not mix completely, leading to strong concentration fluctuations. Notably, with wave heights exceeding 4 m, strong CCN fluctuations and elevated κ values are observed even with winds from the southwest, where the shoreline is more than 4 km away. This indicates that nearshore SSA strongly influences aerosol populations at least 4 km downwind during events with very high waves.

The size distribution of nonvolatile aerosol under shoreline-influencing conditions shows strong increases in number concentrations above 30 nm compared to background periods (Fig. 2D). The increased concentrations of nonvolatile particles suggest that the peak diameter of the nascent SSA particles from shoreline wave breaking ranges from 30 to 100 nm (Fig. 2D and fig. S6), consistent with previously reported nearshore SSA size distributions (*50*) and the abundance of sub-100-nm SSA at coastal sites (*13*, *14*). Aerosols in remote MBL typically exhibit a bimodal distribution, i.e., Aitken and accumulation modes with a Hoppel minimum between them (*51*). The presence of nearshore SSA may notably alter observed ambient aerosol size distribution, obscuring this bimodal characteristic.

### Decoupling of nearshore SSA generation from local wind speed

Previous studies have pointed out that nearshore SSA emission flux can be expressed as a function of wave energy dissipation, which scales with incoming wave height (*29*, *31*, *52*). At any given location, wind waves and swell waves can coexist, with their combined energy determining the total wave height. Our analysis of ocean waves and meteorological parameters in the ENA reveals that high waves are generally dominated by strong swell waves that follow cold air outbreaks. This dominance of swell waves explains why total wave energy and nearshore SSA production are usually decoupled from local wind speed.

**Fig. 3.**
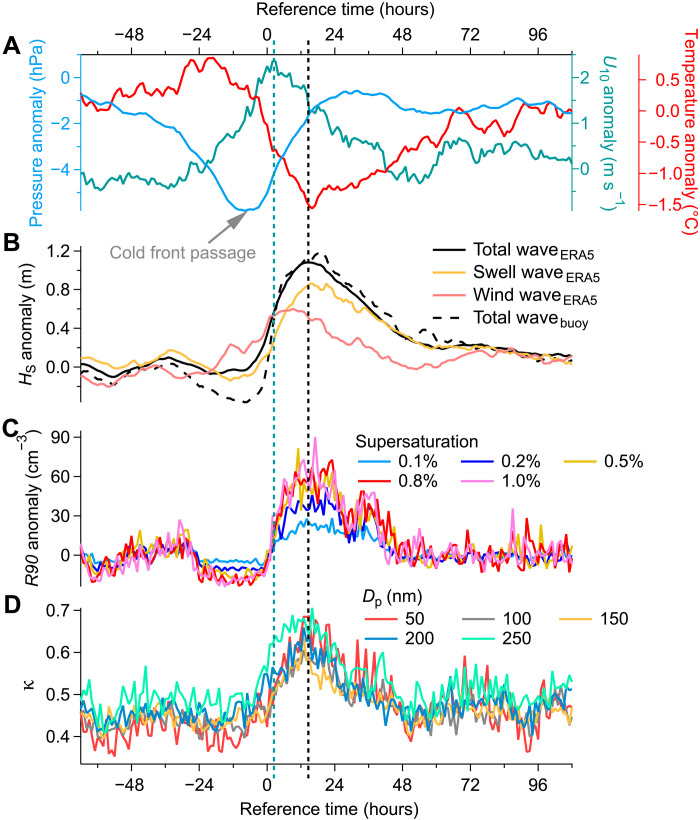
The dominant role of strengthened swell waves associated with cold air outbreaks on nearshore SSA emission in the ENA. We identified a total of 44 cold air outbreak events with rapid *H*_S_ increase during the ACE-ENA campaign, and this figure shows the evolutions of relevant parameters averaged over these events. These parameters include (**A**) anomalies of surface pressure, *U*_10_, and air temperature; (**B**) the anomalies of *H*_S_ corresponding to total waves, wind waves, and swell waves from ERA5, and total waves from buoy measurements; (**C**) anomalies of *R90*; and (**D**) aerosol κ values. The calculations of anomalies are detailed in the Supplementary Text. The reference time of 0 hour represents the moment when total *H*_S_ begins the rapid increase following the passage of cold front. The teal and black vertical dashed lines denote the peak time of the anomalies of *U*_10_ and total wave height, respectively.

The onset of more than half of the high-wave events near the ENA site shows a characteristic pattern (fig. S7): a sudden drop in air temperature, a shift from decreasing to increasing air pressure, an increase in carbon monoxide (CO) mixing ratio, an increase in average latitude along the 10-day air mass backward trajectory, and a notable time fraction of backward trajectory spent over North America. These changes indicate the passage of cold fronts, which primarily originate from high-latitude North America. As these cold air outbreaks move eastward across the North Atlantic, they generate strong wind waves through increased surface wind speeds. The strong wind waves eventually transform into swell waves and propagate outward (fig. S8). At the ENA site, local wind speed typically peaks within 12 hours after a cold front passage and then steadily declines (Fig. 3A). As wind speed decreases, the reduced energy input causes wind wave height to rapidly decrease (Fig. 3, A and B). However, the aged swell waves propagating from stronger storms north of the island arrive later during these waning-wind conditions and dominate the total wave energy (figs. S8 and S9), causing total wave height to become decoupled from local wind speed (Fig. 3B and fig. S10). Further details of this large-scale process are provided in the Supplementary Text.

On average, the peak in total wave height occurs ~12 hours after the peak in local *U*_10_. The timing of maximum CCN fluctuation and aerosol κ value aligns with this peak in total wave height (Fig. 3), further confirming that nearshore SSA production, governed by wave energy rather than local wind speed, is the primary driver of the observed fluctuations and SSA enhancement. During high-wind conditions, wind waves can dominate total wave height (fig. S10) and also produce high nearshore SSA fluxes, leading to positive but weak correlations of *R90* and κ with local wind speed (fig. S4, A and C). However, such wind wave-dominated cases represent only 18.5% of all high-wave periods (*H*_S_ > 2 m).

### Strong impacts of nearshore SSA on coastal CCN and PM

**Fig. 4.**
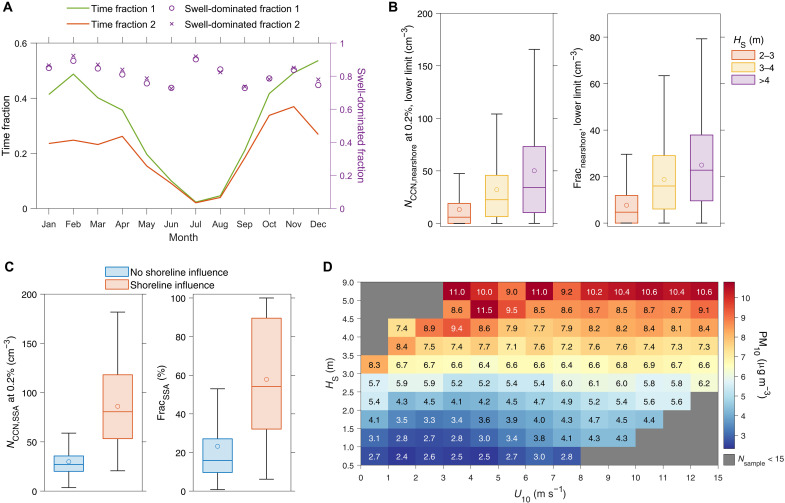
The strong impact of shoreline wave breaking on aerosol population observed at the ENA site. (**A**) Monthly climatologies of the time fraction of shoreline-influencing periods from 2013 to 2023 and the fraction of time with swell wave height exceeding wind wave height during those periods (swell-dominated fraction). The suffixes “1” and “2” denote the results based on two different criteria for identifying the shoreline-influencing periods, which provide estimates of the upper and lower bounds of the time fraction (see Materials and Methods for more information). (**B**) The estimated absolute and relative contributions of nearshore SSA to observed CCN concentrations during the shoreline-influencing periods grouped by different *H*_S_ ranges. The estimation is based on the enhancement of the fluctuation and only represents a lower limit. (**C**) The comparisons of *N*_CCN,SSA_ and Frac_SSA_ between the periods without and with strong shoreline influence. Here, *N*_CCN,SSA_, derived from measured size-resolved particle hygroscopicity and aerosol size distribution, represents the concentration of SSA particles serving as CCN, and Frac_SSA_ represents the fractional contribution of *N*_CCN,SSA_ to the total CCN. For [(B) and (C)], the shoreline-influencing periods correspond to the stricter criterion (criterion 2). The boxes represent upper and lower quartiles, the horizontal lines and circles represent the median and mean values, and the whiskers represent the minimum and maximum values within 1.5 times the interquartile range from the lower and upper quartiles. (**D**) Average PM_10_ concentrations in different *H*_S_ and *U*_10_ ranges. The data outside the periods when the ENA site is downwind of the shoreline wave breaking area (i.e., wave and wind directions meet criterion 1) are not included in (D). The *H*_S_ data in this figure are from ERA5.

To identify periods with strong shoreline wave breaking influence (referred to as shoreline-influencing periods), we established criteria on the basis of the observed dependence of CCN fluctuation and aerosol hygroscopicity on three key parameters: *H*_S_, wave direction, and wind direction. These criteria require both that total wave height exceeds a specific threshold and that the ENA site is downwind of areas where waves break at the shoreline (details in Materials and Methods). Using these criteria, we find that shoreline-influencing periods occur at least 20% and possibly more than 40% of the time from October to April (Fig. 4A). The more frequent occurrence in winter follows the seasonal variation in *H*_S_ (fig. S9A). Most strong waves approaching the northern shoreline propagate from the northwest (fig. S9B) and are minimally affected by refraction and diffraction from Graciosa Island and other islands nearby. Swell waves dominate the total wave height in more than 70% of the shoreline-influencing periods throughout the year (Fig. 4A).

The contribution of nearshore SSA to the CCN population was estimated from the observed *N*_CCN_ fluctuation and represents a lower limit (see Materials and Methods). During the frequent shoreline-influencing events, nearshore SSA substantially contributes to observed *N*_CCN_, and the contribution increases with *H*_S_ (Fig. 4B). On average, the lower limit of the contribution surpasses 50 cm^−3^ and represents >30% of *N*_CCN_ when *H*_S_ exceeds 3.5 m and *U*_10_ is below 7 m s^−1^ (fig. S11). Using a similar approach to previous studies (*13*, *14*), we also quantified the concentration of SSA particles serving as CCN (*N*_CCN,SSA_) based on size-resolved hygroscopicity and aerosol size distribution (see Materials and Methods). Similar to the CCN fluctuation and aerosol hygroscopicity, *N*_CCN,SSA_ strongly increases with *H*_S_ at any given *U*_10_ level while showing no trend with *U*_10_ at nearly all *H*_S_ levels (fig. S11). This indicates that, under high-wave conditions, SSA-derived CCN observed at this site is dominated by SSA generated near shoreline. During background periods, *N*_CCN,SSA_ averages 29.8 cm^−3^ (interquartile range, 20.1 to 35.7 cm^−3^), contributing below 30% of total CCN 78% of the time. Conversely, during shoreline-influencing periods, *N*_CCN,SSA_ (average, 86.1 cm^−3^; interquartile range, 53.2 to 118.1 cm^−3^) triples, representing more than 50% of total CCN 54% of the time (Fig. 4C). Throughout the year, nearly all aerosols observed with sea salt–like hygroscopicity (κ > 1) below 200 nm and/or high *N*_CCN,SSA_ (>100 cm^−3^ at 0.2% supersaturation) are associated with nearshore SSA generation (fig. S12). Therefore, the concentration of SSA and its contribution to CCN population in remote MBL will likely be overestimated if nearshore generation of SSA is not taken into account and all SSA population observed at a coastal site is attributed to open-ocean wave breaking. Shoreline wave breaking also greatly enhances aerosol mass concentration, as previously revealed (*31*, *32*), and, again, this enhancement exhibits a much stronger dependence on *H*_S_ than *U*_10_ (Fig. 4D). The concentration of aerosol with aerodynamic diameter less than 10 μm (PM_10_) increases by over 4 μg m^−3^ and more than doubles when *H*_S_ increases from less than 2 m to above 4 m at many *U*_10_ levels (Fig. 4D). These pronounced increases can substantially affect coastal air quality.

**Fig. 5.**
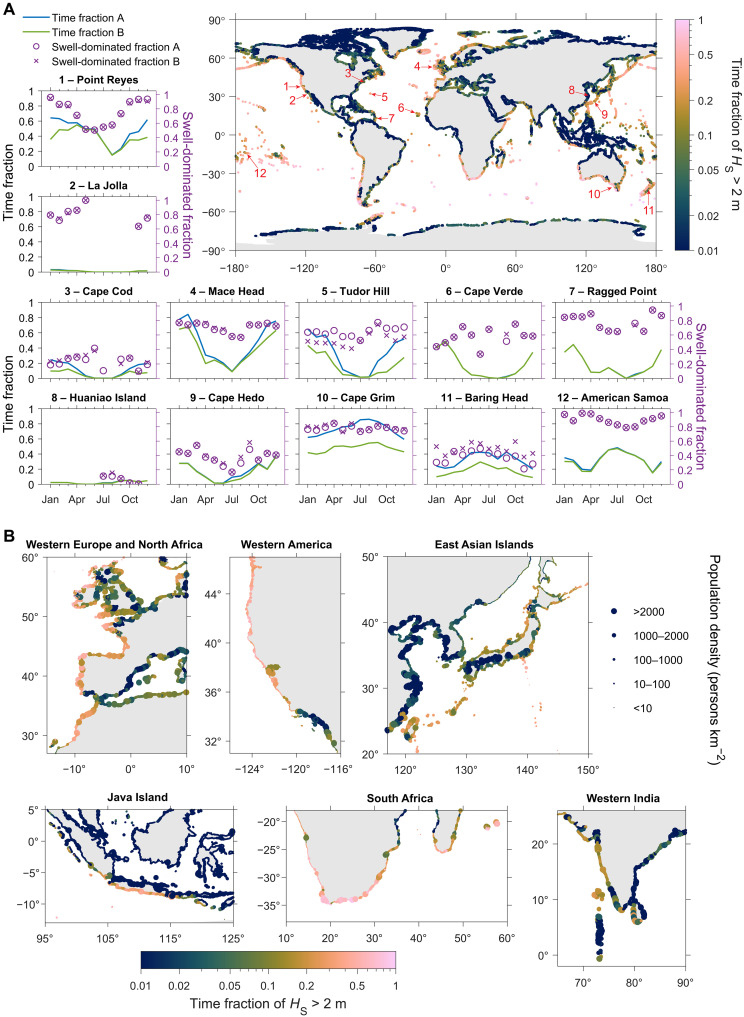
Global distribution of coastal wave height and regions potentially with strong impacts of nearshore SSA production. (**A**) The time fraction of high-wave periods (*H*_S_ > 2 m) from 2013 to 2022 along global coastlines. The data missing at Antarctic coastlines are due to yearlong sea-ice coverage. For the 12 representative aerosol observation stations, the suffix “A” corresponds to high-wave periods (*H*_S_ > 2 m). For suffix “B,” in addition to the high-wave conditions, the wave and wind directions are also considered to ensure the station is downwind the shoreline area with incoming waves. Detailed information of the stations and corresponding criterion B are listed in table S3. The swell-dominated fraction refers to the fraction of time when total wave height is dominated by swell wave during those high-wave periods. (**B**) Enlarged maps showing the time fraction of high-wave events and population density in six hotspot regions with both frequent strong waves and large coastal population. The *H*_S_ data in this figure are from ERA5.

Although the ENA site is located on a small island, the physical mechanisms that drive nearshore wave breaking and SSA generation are fundamentally similar to those at continental coastlines. However, different coastal topography and seafloor slopes may substantially affect the spatial extent and intensity of shoreline wave breaking (*52*). The ENA site is close to the steep rocky shoreline of the Graciosa Island. To further examine the generality of the strong nearshore SSA impact under high-wave conditions, we also investigated the impact of nearshore SSA generation from flat beach shorelines using measurements at Cape Cod and Point Reyes located on the eastern and western coasts of the US, respectively (see Materials and Methods). The measurement sites are 160 m (Cape Cod) and 1160 m (Point Reyes) from the shoreline. Both sites showed strong increases in aerosol concentration and fluctuation when *H*_S_ exceeds 1.5 to 2 m with onshore winds (fig. S13). The much stronger dependence on *H*_S_ than on *U*_10_ further confirms that the increased aerosol concentrations are due to shoreline wave breaking (figs. S14 and S15). Given the strong impact on both *N*_>10_ and PM_10_ concentration, dominated by ultrafine and coarse particles, respectively, nearshore SSA is expected to contribute substantially to the CCN population as well under high-wave conditions. At both sites, average PM_10_ concentration increased by ~10 μg m^−3^ or more as *H*_S_ increased from below 1.5 m to above 3.0 m at many *U*_10_ ranges (fig. S15), and such increase is even stronger than that observed at the ENA site. High-wave events in Cape Cod and Point Reyes are mostly dominated by wind waves and swell waves, respectively (Fig. 5A). The results from the three sites indicate that intense nearshore SSA emissions under strong-wave conditions can substantially enhance coastal aerosol populations across a wide size range from 10 nm to several micrometers, and such phenomenon is common and independent of wave type, shoreline characteristics, or whether the coast is island or continent based.

## DISCUSSION

**Fig. 6.**
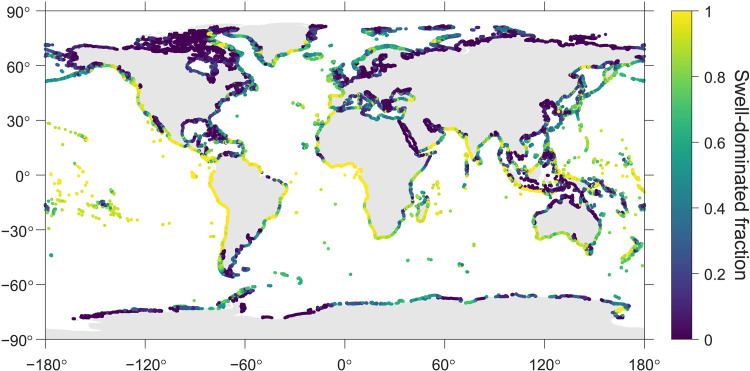
The fraction of time when total wave height is dominated by swell wave (swell-dominated fraction) during high-wave periods from 2013 to 2022 along global coastline. The wave height data are from ERA5.

Global wave statistics show that strong nearshore waves occur frequently along a large portion of global coastlines, particularly in mid-latitudes and around small mid-ocean islands (Fig. 5A). The aerosol population near these coastlines is likely to be affected by strong shoreline wave breaking for a substantial amount of time. In addition, because swell waves contribute 50 to 100% of total wave energy across most open oceans (*22*, *23*), many coastlines bordering open oceans are predominantly affected by swell waves during high-wave periods (Fig. 6). This swell-dominated time fraction is especially high (>0.9) along many tropical coastlines, while, in Northern Hemisphere mid-latitude regions, swell dominance tends to be more prevalent along western continental margins compared to their eastern counterparts. These patterns align with previous results showing extremely high swell probabilities in equatorial oceans (i.e., swell pools) and the eastward propagation of swell waves in extratropical areas (*22*, *23*). The widespread strong production of nearshore SSA and its decoupling from local wind speed under swell-dominated conditions, as shown in this study, have important implications for our understanding of SSA’s environmental effects, particularly in the context of observations and modeling approaches.

First, many studies on SSA emissions, properties, and climate effects are based on coastal observations (*13*, *14*, *38*, *39*). The observed SSA properties at coastal sites, such as size distributions, may not accurately represent open-ocean SSA due to different wave breaking mechanisms, which requires further investigation. If coastal observations are affected by SSA generation from shoreline wave breaking, then extrapolating results to open oceans without considering this impact can greatly overestimate both SSA concentration and its contribution to CCN population in remote marine environment, and, consequently, the effects of SSA on clouds and climate. In addition, the derived relationships between SSA emission fluxes or concentrations on environmental variables (e.g., wind speed) are likely not applicable to open oceans, even if the wave energy is dominated by wind waves. Although many SSA source functions in global models are developed on the basis of open-ocean measurements or laboratory simulations, the evaluation and validation of these functions usually include coastal observations (*41*, *42*), which may introduce substantial biases (*41*).

To further explore the potential impact of nearshore SSA on coastal measurements, we analyzed wave statistics near 12 representative coastal atmospheric observatories. The results reveal that high-wave periods occur during more than half the time in certain seasons at several stations, e.g., Point Reyes, Mace Head, Turdor Hill, and Cape Grim, and swell waves often play dominant roles (Fig. 5A). This analysis provides a qualitative estimate of how frequently substantial nearshore SSA influences might occur. The magnitude of influence also depends on other factors like ocean bathymetry, coastal topography, station location (distance to the shoreline and altitude), and micrometeorology (*24*, *29*, *36*). A comprehensive understanding necessitates detailed analyses at specific stations. The methods developed in this study for identifying the impact of nearshore SSA (i.e., examining whether enhanced aerosol fluctuation, concentration, and SSA signatures such as high hygroscopicity and nonvolatile fraction occur with high waves and onshore winds) can be readily applied to long-term measurements at other coastal sites, which helps understand whether and when the coastal measurements represent open-ocean conditions.

Second, beyond its influence on aerosol observations, the substantial contribution of nearshore SSA to coastal CCN population (and potentially giant CCN as well) suggests a need for further investigation into its effects on fog formation, cloud development, and precipitation in coastal regions. Previous modeling studies (*28*, *29*, *44*) and lidar observations (*33*, *34*) have shown that the SSA generated along the shoreline can retain more than 10% of its original concentration after being transported 20 km, and the plume can evolve to several hundred meters in height. A remote sensing study at Nauru found that nearshore SSA generation, accompanied by an upward air motion after propagating into the warmer island, leads to the formation of frequent cloud trails extending for tens of kilometers (*53*). Understanding these effects in coastal regions requires models that incorporate a wave energy-based parameterization to accurately represent nearshore SSA emission, which depends on wave energy dissipation and is often decoupled from local wind speed. However, many current regional models neglect nearshore SSA generation (*54*, *55*) and likely underestimate the overall coastal CCN population. While a few modeling studies account for enhanced SSA emissions from the surf zone, they either assume fixed surf width and whitecap fraction (*56*, *57*), which cannot accurately capture the spatial and temporal variations, or use parameterizations based on local wind speed (*58*, *59*), which is not suitable for the widespread regions where swell waves dominate (Fig. 6). Previous studies noted that nearshore SSA generation can be decoupled from local wind speed due to the role of swell waves (*20*, *21*); here, we show that such decoupling is likely to be frequent over large coastal regions globally (Fig. 6). Coupling a high-resolution atmospheric model with a high-resolution coastal ocean wave model as well as a valid wave energy-driven parameterization for nearshore SSA flux can allow better simulations of the elevated CCN concentration and subsequent environmental effects in coastal regions. Direct aerosol flux measurements [e.g., eddy covariance methods; (*30*)] across various shore types, combined with simultaneous characterization of wave fields to calculate wave energy dissipation, can facilitate developing such parameterization for nearshore SSA emissions.

Third, nearshore SSA generation may strongly affect the coastal atmospheric chemistry and air quality. Compared to remote oceans, coastal waters are typically more enriched with pollutants and algal biomass. A wide range of chemical and biological pollutants in coastal regions, such as persistent organic pollutants, micro- and nanoplastics, harmful algal and bacteria, and biogenic toxins, can be transferred into the atmosphere via SSA emission and potentially pose health impact to nearby populations (*45*–*48*, *60*, *61*). For example, human respiratory irritations associated with SSA during red tide events have been reported by several studies (*61*–*63*). Additionally, the release of reactive halogens from SSA and their influence on ozone photochemistry could also affect human health (*59*, *64*). Moreover, SSA can strongly contribute to aerosol extinction in coastal regions, thereby reducing visibility (*65*). Our results show that, during high-wave periods, nearshore SSA can enhance PM_10_ concentration by >10 μg m^−3^ more than 1 km downwind from the shoreline. Therefore, in the coastal regions with both high population density and frequent strong waves, such as Western Europe, North Africa, Western America, East Asian Islands, Southern Java Island, South Africa, and Western India (Fig. 5B), nearshore SSA may pose strong impacts on human health and visibility for large populations, and such impacts have been largely overlooked. Notably, these regions are usually dominated by swell waves (Fig. 6). Due to the same reason as discussed above, current regional air quality models, which do not incorporate a wave energy-driven parameterization, cannot accurately capture the influences of nearshore SSA on coastal air quality and associated health impact on coastal communities. In addition, more studies of the chemical and biological composition of nearshore SSA are also needed, particularly in regions with polluted seawater. With a changing climate, it is also important to evaluate the future trend of the impact from nearshore SSA, alongside sea-level rise and changes in both storm intensity and frequency (*66*).

In summary, strong nearshore SSA production during high-wave periods, driven by incoming waves lapping the shoreline and wave breaking in the surf zone, greatly enhances both aerosol number and mass concentrations in downwind areas. The contribution of SSA to CCN and aerosol mass concentration can increase by over threefold and exceed 10 μg m^−3^, respectively. The wave energy in many coastal regions is dominated by remotely generated swell waves, which typically do not break in deep open oceans but break intensely in shallow coastal waters when interacting with the seafloor near the shoreline. Under frequent swell-dominated conditions, the production of nearshore SSA is decoupled from wind waves and local wind speeds. Given its different mechanism from open-ocean SSA generation, strong impact on coastal aerosol population, and prevalence along a large fraction of global coastlines, nearshore SSA generation must be considered both when using coastal measurements to study the properties and climate effects of SSA over open oceans and when modeling the coastal aerosol population and its environmental impacts. Coastal observations under the influence of strong shoreline wave breaking are not representative of open-ocean conditions. Therefore, extrapolating results to open oceans without taking it into account may significantly overestimate SSA concentration and its contribution to CCN in remote marine environments and, consequently, the impact of SSA on clouds and climate. In addition, without properly representing nearshore SSA generation, models likely underestimate aerosol population and its impact on air quality and cloud development in coastal regions.

## MATERIALS AND METHODS

### Aerosol measurements near the shoreline

#### Observation sites

The main observation site of this study is the ENA observatory (39.09°N, 28.03°W, 30 m above mean sea level) located on Graciosa Island in the Azores, Portugal (fig. S1). The ENA observatory is a ground site established by the US Department of Energy Atmospheric Radiation Measurement (ARM) Climate Research Facility in 2013 (*67*). The Graciosa Island has a length of ~10 km and a width of ~7 km. The observatory is located near the northernmost edge of the island, with the closest distance to the shoreline being 470 m to the north and the farthest distance in the southeast direction being ~11 km. Aerosol measurements at two additional sites near the beach shorelines are also used to further demonstrate the ubiquitousness of the strong impact of nearshore SSA. These two sites are located in Cape Cod (42.03°N, 70.05°W, 43 m above mean sea level) and Point Reyes (38.09°N, 122.96°W, 24 m above mean sea level) on the eastern and western coasts of the US, respectively (fig. S1). The distances from the nearest shoreline are 160 m for Cape Cod and 1160 m for Point Reyes, and the corresponding onshore wind direction sectors are [320°, 360°] ∪ [0°, 150°] and [200°, 360°], respectively.

#### Aerosol measurements

A suite of instruments has been deployed at the ENA site to comprehensively characterize the properties of atmospheric aerosols since October 2013 (*67*). The total number concentration of aerosol particles larger than 10 nm (*N*_>10_) was measured by a condensation particle counter (CPC) with a time resolution of 1 s. CCN concentrations (*N*_CCN_) at five different supersaturation levels were measured by a CCN counter. The supersaturation level cycled through 0.1, 0.2, 0.4 or 0.5, 0.8, and 1.0% and changed every 10 min. The time resolution of raw data is 1 s. Aerosol scattering coefficients (*b*_sca_) were measured by a three-wavelength Integrating Nephelometer with a time resolution of 5 s, and the *b*_sca_ values corresponding to green light (wavelength, 550 nm) were used in this study. The sample cut size for the Nephelometer measurements alternated between 1 and 10 μm twice every hour (*67*). The measured *b*_sca_ values were also used to estimate the PM_10_ mass concentration, and the details are provided in the last subsection of Materials and Methods. Aerosol number size distribution from 60 nm to 1 μm was measured by an ultrahigh-sensitivity aerosol spectrometer (Droplet Measurement Technologies) with a time resolution of 10 s. To characterize the hygroscopicity of aerosols, a humidified tandem differential mobility analyzer (HTDMA) was used to measure the size-resolved hygroscopic growth factor (GF) at 80 or 85% relative humidity (RH). The measurement cycled through dry diameters of 50, 100, 150, 200, and 250 nm, and it took 6.2 or 11.2 min to obtain the GF spectrum for one dry size. During the Aerosol and Cloud Experiments in ENA (ACE-ENA) campaign from June 2017 to August 2018 (*68*), a scanning mobility particle size (SMPS) was deployed at the ENA site to characterize the dry aerosol number size distribution from 10 to 470 nm (RH < 25%). The measurement alternated every 4 min between ambient aerosol samples and the residual aerosols after being heated to 300°C in a thermal denuder (*69*). Sea salt is nonvolatile at 300°C and can survive the thermal denuder. The data obtained by SMPS were corrected for diffusional losses in the sampling line and multiple charging (*70*, *71*).

The ARM’s Mobile Facility was deployed on Cape Cod during the Two-Column Aerosol Project field campaign from 2012 to 2013 and at Point Reyes during the Marine Stratus Radiation Aerosol and Drizzle field campaign in 2005. *N*_>10_ and *b*_sca_ were measured by CPC and Nephelometer, respectively, with a time resolution of 1 min. Details of aerosol measurements at the three sites and the periods when valid measurements are available are listed in table S1.

#### Derivation of aerosol hygroscopicity parameter

Based on the measured GF distribution, the hygroscopicity parameter (κ) under subsaturated conditions was derived (*72*, *73*)

$$\kappa =\left({\text{GF}}^{3}-1\right)\left[\frac{\text{exp}\left(\frac{A}{D_{d}\text{GF}}\right)}{\text{RH}}-1\right]$$ (1)

$$A=\frac{4\sigma_{s/a}M_{w}}{\mathrm{RT}\rho_{w}}$$ (2)

where *D*_d_ is the particle dry diameter, $\sigma_{s/a}$ is the surface tension of the solution/air interface (here, assumed to be that of pure water, 0.072 N m^−1^), *M*_w_ is the molecular weight of water, *R* is the universal gas constant, *T* is the humid scan temperature, and ρ_w_ is water density. To derive the average κ of ambient hygroscopic aerosols at a specific dry diameter, we first fitted the GF probability distribution spectrum with up to three Gaussian distributions and then derived the κ value corresponding to hydrophilic mode (GF_mode_ > 1.1). If there were more than one hydrophilic mode, then the number-weighted average of the κ values corresponding to the hydrophilic modes were used. Examples illustrating this GF mode fitting procedure and the corresponding results are shown in fig. S16.

#### Ancillary data

During all the aerosol measurement periods, meteorological parameters including air temperature, 10-m wind speed (*U*_10_), and 10-m wind direction were characterized concurrently. The concentration of carbon monoxide (CO) was measured by a CO/H_2_O/N_2_O analyzer (Los Gatos Research Inc.). Regional surface meteorological reanalysis from the Modern-Era Retrospective Analysis for Research and Applications, version 2 (MERRA-2; spatial resolution, 0.5° × 0.625°) (*74*) was used to analyze the large-scale synoptic conditions and processes. It should be noted that, due to differences in surface topography and heat capacity between land and ocean, a systematic bias may exist between the measured *U*_10_ and the *U*_10_ over water, where SSAs are generated. Because of the lack of direct meteorological buoy measurements in the Azores region, we compared *U*_10_ measured at the ENA site with *U*_10_ from MERRA-2 reanalysis. The *U*_10_ measured at the ENA site is highly correlated with MERRA-2 *U*_10_ in the grid cell containing Graciosa Island [coefficient of determination (*R*^2^) = 0.71], showing a small mean absolute difference of 1.87 m s^−1^. This indicates that any bias in *U*_10_ measured at the ENA site is likely minor, and the temporal variations of measured *U*_10_ are consistent with those over water nearby. Therefore, the potential bias in *U*_10_ is not expected to substantially affect the conclusions of this study.

### Quantification of the fluctuation intensity of aerosol concentration

For the CCN measurement at the ENA site, ~600 1-s data points of CCN concentration were obtained in each 10-min window for a specific supersaturation level. Because the supersaturation level inside the instrument may not stabilize at the set point during the first several minutes, we focused on the measurements during the last 5 min of each 10-min window. Among the 300 CCN data points over the 5-min period, the difference between the 95th and 5th percentile values (referred to as *R90*) was used to quantify the short-term fluctuation of CCN concentration.

For the total aerosol number concentration measurements at Cape Cod and Point Reyes, only 1-min time resolution data are available. We first excluded the extremely high concentrations (*N*_>10_ > 3000 cm^−3^) due to the strong perturbation by anthropogenic pollution. Then, we calculated the relative deviation of each 1-min data point compared to the average *N*_>10_ within a 5-min time window (denoted as RD)

$${\text{RD}}_{t}=\frac{\left|N_{>10,t}-\frac{1}{5}\sum_{i=t-2}^{t+2}N_{>10,i}\right|}{\frac{1}{5}\sum_{i=t-2}^{t+2}N_{>10,i}}$$ (3)

where *t* is the time index with a unit of minute. The fluctuation intensity for each hour is then defined as the median of all RD values within the corresponding 1-hour time window. It should be noted that the timescales are different for these two fluctuation metrics (second-scale for CCN measurement at ENA site and minute-scale for total aerosol number concentration measurements at Cape Cod and Point Reyes), but they are both significantly shorter than the typical timescales for the variations of natural aerosol number concentrations in clean environments.

### Ocean wave data

Six directional Waverider buoys (DWR-MkIII, Datawell BV) have been deployed across Azores to continuously monitor ocean wave parameters. These buoys measure the heave and translational motion at a rate of 1.28 Hz and report significant wave height (*H*_S_, defined as the average height of the largest one-third waves in a given wave record), mean wave period, and mean wave direction every 10 min. We primarily used data from 2013 to 2023 from the buoy located ~2.4 km east of Graciosa Island. During periods when data from the Graciosa buoy are unavailable (accounting for 21.3% of the total period), we supplemented with measurements from the buoy located between Faial and Pico Islands. Significant differences in *H*_S_ measurements between the two buoys exist only when waves approach from the east, a direction in which the Faial/Pico buoy is partially blocked by surrounding islands, whereas the Graciosa buoy is not. However, such conditions only account for 8.1% of the time. During the remaining periods, *H*_S_ measurements from the two buoys are in good agreement, with a mean absolute difference of 0.25 m, a linear regression slope of 0.96, and an *R*^2^ of 0.88. Given this consistency, it is reasonable to combine the wave data from these two buoys for the evaluation of ERA5 wave data in the Azores, which is detailed below.

The global ocean wave states corresponding to wind waves, swell waves, and their combination (referred to as total waves in this study) were retrieved from the European Center for Medium-range Weather Forecast (ECMWF)’s Reanalysis v5 (ERA5; hourly, 0.5° × 0.5°) (*75*). In ERA5, ocean wave variables are simulated by the ECMWF wave model (ECWAM), in which the physics is based on the Wave Modeling (WAM) Cycle 4 (*76*) and the wave advection scheme, unresolved bathymetry scheme, and input source/dissipation terms have been updated (*77*). In addition, numerous observations from satellite altimeters were also assimilated. Previous studies have shown that the total *H*_S_ from ERA5 is in good agreements with in situ observations from buoys (*78*). Here, we compared the combined buoy-measured *H*_S_ with the ERA5 total *H*_S_ in the grid cell that contains the entire Graciosa Island. The results show that both the long-term seasonal cycles and short-term variations of observed *H*_S_ are well reproduced by ERA5, and the absolute values of observed *H*_S_ and ERA5 *H*_S_ are highly consistent when waves approach from the north (i.e., when incoming waves are not blocked by any island, fig. S2), which further proves the high accuracy of ERA5 ocean wave data. When waves approach from certain directions (wave direction ∈ [150°, 315°] for the Graciosa buoy and wave direction ∈ [45°, 300°] for the Faial/Pico buoy), the buoys cannot accurately capture the properties of incoming waves due to the blockage by nearby islands. Consequently, the observed *H*_S_ is substantially lower than that from ERA5, which represents the regionally average wave state in a grid cell. Therefore, all *H*_S_ and wave direction data used in our quantitative analyses are from ERA5. Buoy-measured *H*_S_ data are presented only in Figs. 1 and 2 and fig. S3 for time-series illustration.

### Criteria for identifying the periods with strong influences of shoreline wave breaking

The general conditions when coastal measurements are strongly influenced by shoreline wave breaking include that (i) there are strong incoming ocean waves leading to intensive wave breaking near the shoreline, and (ii) the measurement site is downwind of the wave-breaking area. Therefore, we established the criteria by considering the impacts of wave direction on the location of shoreline wave breaking, *H*_S_ on wave breaking intensity, and wind direction on whether the measurement site is downwind of the wave-breaking area or not. The characteristics of observed CCN fluctuation and aerosol hygroscopicity were investigated to identify the appropriate thresholds for the criteria.

For the ENA site, we established two different criteria. For the first one (criterion 1), the wave direction was segregated into seven sectors based on the shape of the Graciosa Island. Each wave direction sector corresponds to a sector of shoreline where wave breaking occurs. For each wave direction sector, the dependence of *R90* and κ on wind direction and *H*_S_ was investigated. The range of wind direction was further segregated into one to three sectors based on the distance from the shoreline to the site. Wind direction ranging from 110° to 210° was excluded because of the blockage of the nearby hills, which is confirmed by the lack of observed influence by nearshore SSA (i.e., elevated *R90* and κ). The *H*_S_ threshold was then determined for each wind direction sector to capture all evident influences by nearshore SSA. Figure S17 shows an example illustrating the procedure of establishing the criterion. Detailed information of the established criterion 1 is listed in table S2. Some periods identified on the basis of this criterion show no obvious influence by shoreline SSA, especially when winds come from the southwest. Therefore, the calculated time fraction of shoreline-influencing periods based on criterion 1 can be regarded as the upper limit. For the second criterion (criterion 2), only the periods when both ocean waves and winds are from the north (wave direction ∈ [280°, 360°] ∪ [0°, 90°] and wind direction ∈ [280°, 360°] ∪ [0°, 100°]) are considered. The distance from shoreline to the site is less than 1.8 km under this wind direction sector. The threshold of *H*_S_ is set as 2 m. During periods identified by criterion 2, measurements at the ENA site show strong influences by shoreline wave breaking. The time fraction of shoreline-influencing periods based on this stricter criterion likely represents the lower limit. We note that these criteria correspond to “strong influences” of shoreline wave breaking. For the periods not meeting these criteria, e.g., when wave direction and wind direction meet the criteria but the *H*_S_ is lower than the threshold, the influence of shoreline wave breaking may still exist, albeit relatively weak. Such weak influences are suggested by the slight enhancements of *N*_CCN,SSA_ and PM_10_ concentration with increasing *H*_S_ below 2 m (fig. S11 and Fig. 4D).

### Estimation of the contribution of nearshore SSA to CCN and total aerosol number (lower limit)

In each 5-min CCN measurement window during shoreline-influencing periods, the emission flux of nearshore SSA can fluctuate very strongly. Hence, we assume the instantaneous nearshore SSA concentration can drop to zero at certain times, i.e., the minimum of observed CCN concentration in the 5-min time window is not affected by nearshore SSA and represents the background CCN concentration. Because the fluctuation of background CCN concentration is significantly lower than that due to the shoreline wave breaking, the average contribution of nearshore SSA to CCN within a 5-min time window during shoreline-influencing periods (denoted as *N*_CCN,nearshore_) can be estimated by the following equations

$$N_{\text{CCN},\text{nearshore}}=∆N_{\text{CCN}}-∆N_{\text{CCN},\text{background}}$$ (4)

$$∆N_{\text{CCN}}=N_{\text{CCN},\text{mean}}-N_{\text{CCN},\text{min}}$$ (5)

*N*_CCN,mean_ and *N*_CCN,min_ represent the mean and minimum CCN concentrations in the 5-min measurement window, respectively. Δ*N*_CCN,background_ is the Δ*N*_CCN_ value when the influence of nearshore SSA is minimal. In this study, the median Δ*N*_CCN_ under background conditions within the 1-month period centered on the targeted time window is used. The background conditions correspond to the periods when criterion 1 is not satisfied.

It is important to note that the lowest nearshore SSA emission flux may not be zero during shoreline-influencing periods. Additionally, partial mixing of nearshore SSA plumes occurs as the plumes travel from the shoreline to sampling site, especially under high-wind conditions. As a result, the observed minimum CCN concentration probably include some contribution from nearshore SSA. Therefore, the approach above likely underestimates the contribution of nearshore SSA to CCN, representing a lower limit.

A similar method was also applied to estimate the lower limit of the contribution of nearshore SSA to total aerosol number concentration at Cape Cod. Because the raw data were recorded with 1-min time resolution, the sub-minute fluctuation cannot be directly obtained. We added one step to first estimate 1 Hz Δ*N*_>10_ in 5-min time window using the recorded 1-min data. The details are presented in the Supplementary Text.

### Quantification of the contribution of SSA to CCN

The contribution of SSA particles to CCN was quantified by combining the hygroscopic GF measured by HTDMA and the particle number size distribution measured by SMPS. Here, the SSA particles are identified by the ultrahigh hygroscopicity of inorganic sea salt. SSA may exhibit lower hygroscopicity due to the internal mixing with organic compounds (*79*, *80*), but some studies found that the κ value for SSA remains high even with a large organic content (*81*). For this study, given the oligotrophic nature of remote mid-latitude Atlantic seawaters and the occurrence of high waves primarily during the cold season with weak biological activity, we expect that SSAs are dominated by sea salt at the ENA site. The GF probability distribution measured by HTDMA was first converted to κ probability distribution based on Eq. 1. We used a κ threshold of 0.67 to separate SSA mode from non-SSA mode (*13*). The number fraction of SSA particles (*F*_SSA_) was then derived by integrating the κ probability distribution with κ > 0.67 at each of the 5 dry diameters (i.e., 50, 100, 150, 200, and 250 nm). The particle number size distribution between 10 and 470 was measured by a SMPS. Between 50 and 250 nm, a size-dependent *F*_SSA_ was derived by linearly interpolating the *F*_SSA_ values at the five diameters. Below 50 nm and above 250 nm, *F*_SSA_ was assumed to have the same values at 50 and 250 nm, respectively. The product of the size-dependent *F*_SSA_ and measured particle number size distributions provides the dry size distributions of SSA from 10 to 470 nm.

At a specific supersaturation level (ss), the critical dry diameter (*D*_crit_) of SSA that can serve as CCN is given by κ-Köhler theory (*72*)

$$D_{\text{crit}}=\sqrt[{3}]{\frac{4A^{3}}{27\kappa {\text{ln}}^{2}\left(1+\text{ss}\right)}}$$ (6)

Here, a value of 1.12 was used to represent κ of SSA (*13*, *72*). *A* is defined by Eq. 2. The values of *D*_crit_ corresponding to supersaturation levels of 0.1, 0.2, 0.5, 0.8, and 1.0% are 108, 68, 37, 27, and 23 nm, respectively. The concentration of the CCN contributed by SSA (*N*_CCN,SSA_) was then obtained by integrating the number size distribution of SSA particles larger than *D*_crit_. We note that there may be substantial uncertainties in derived *F*_SSA_ values below 50 nm and above 250 nm, as HTDMA measurement are only available from 50 to 250 nm. However, the uncertainty between 10 and 50 nm does not affect the derived *N*_CCN,SSA_ values at the lowest two supersaturation levels because the corresponding *D*_crit_ is larger than 50 nm. The uncertainty in *F*_SSA_ between 250 and 470 nm has minimal impact due to the low particle number concentration in this size range.

### Estimation of aerosol mass concentration

We assume that the mass concentration of aerosol with aerodynamic diameter less than 10 μm (PM_10_) is dominated by SSA particles under clean marine conditions, i.e., over remote oceans or over coastal sites under onshore-wind conditions (*10*). The PM_10_ mass concentration was then estimated on the basis of the relationship between inorganic SSA scattering coefficient and mass concentration given by the Interagency Monitoring of Protected Visual Environments (IMPROVE) algorithm (*82*)

$$b_{\text{sca},\text{SSA}}=1.7\times f_{\text{SSA}}\left(\text{RH}\right)\times M_{\text{SSA}}$$ (7)

where *b*_sca,SSA_ is the SSA scattering coefficient, and the PM_10_ *b*_sca_ measured by Nephelometer was used in this study. The value of 1.7 represents the dry sea-salt mass scattering efficiency. *f*_SSA_(RH) is the enhancement ratio of SSA scattering due to hygroscopic growth as a function of RH, as described by Pitchford *et al.* (*82*). *M*_SSA_ is the dry mass concentration of SSA, which corresponds to the estimated PM_10_ mass concentration in this study.

It is worth noting that other aerosol components originating from non-sea spray sources (e.g., dust, secondary formation, and biomass burning) may occasionally make substantial contributions to PM_10_ mass concentration, potentially resulting in biases in our estimation. On the other hand, our aim is to evaluate the impacts of *H*_S_ and *U*_10_ on PM_10_ mass concentration. Because the production of these non-sea spray components is expected to be independent of *H*_S_ or *U*_10_, the variations of their concentrations will likely be averaged out when the data are grouped into different *H*_S_ and *U*_10_ bins. Therefore, the statistical differences of estimated PM_10_ concentrations under different *H*_S_ and *U*_10_ conditions can be reasonably attributed to SSA. In addition, the variations in SSA size distribution and the presence of primary organics in SSA can also lead to the deviation in the actual mass scattering efficiency and its RH dependence function from those used in the calculation. But these uncertainties are not expected to strongly affect the main conclusions of this study. It also should be noted that the PM_10_ cutoff size corresponds to the wet aerodynamic diameter under ambient RH. The equivalent dry aerodynamic diameters are mostly between 4.5 and 5.7 μm (accounting for >90% of the data). Because the inlet cutoff for routine PM measurements in air quality monitoring networks is also corresponding to wet aerodynamic diameter under ambient conditions, we did not apply further adjustment to the particle size range.

## Supplementary Materials

### This PDF file includes:

Supplementary Text Figs. S1 to S18 Tables S1 to S3 References
