# Supplementary material for: Shoreline wave breaking strongly enhances the coastal sea spray aerosol population: Climate and air quality implications
Source: Sci Adv. 2025 Aug 27;11(35):eadw0343. doi: 10.1126/sciadv.adw0343 (PMC12383254; doi:10.1126/sciadv.adw0343)
Supplement: Supplementary file 1 — Supplementary Text Figs. S1 to S18 Tables S1 to S3 References [file sciadv.adw0343_sm.pdf]

Science Advances
AAAS

Supplementary Materials for

Shoreline wave breaking strongly enhances the coastal sea spray aerosol population: Climate and air quality implications

Shengqian Zhou *et al.*

Corresponding author: Jian Wang, jian@wustl.edu

Sci. Adv. **11**, eadw0343 (2025)
DOI: 10.1126/sciadv.adw0343

This PDF file includes:

Supplementary Text
Figs. S1 to S18
Tables S1 to S3
References

Supplementary Text

Local anthropogenic pollution and screening of CCN and SMPS measurements at the ENA site

About 4,000 people reside on Graciosa Island, and major residential areas are located to the southeast of the ENA site. There is also an airport adjacent to the ENA site, receiving ~670 flights per year (*49*). A port is located on the southeastern side of Graciosa Island and used for inter-island transport within the Azores. Therefore, the human activities on the island, such as motor vehicle traffic, aircraft landing/taking off, and ship operations, emit abundant aerosol particles and occasionally affect the aerosol measurements (*49, 83*). Since these anthropogenic aerosols are mostly associated with combustions and experience little atmospheric ageing before reaching the site, they are dominated by small particles with diameters down to 10 nm (the lower limit of SMPS) and enriched with less-hygroscopic organics and black carbon. Consequently, while total number concentration of aerosols larger than 10 nm ($N_{>10}$) exhibits strong enhancements and fluctuations when the ENA site is influenced by local anthropogenic pollution, measured CCN concentration (N_{CCN}) and aerosol scattering coefficient are much less affected, as shown by the case from 26 to 27 February 2018 in Fig. 1A. Occasional spikes in N_{CCN} due to local anthropogenic pollution are more frequent at supersaturation levels above 0.5%, and they were identified using the following criteria:

$$\frac{R90_t - R90_{\text{1-day median}}}{R90_{\text{1-day 75-percentile}} - R90_{\text{1-day median}}} > 5, \text{ or}$$

$$\frac{R90_t}{R90_{\text{12-hour median}}} > 3, \text{ or}$$

$$N_{\mathrm{CCN},t} > \text{threshold}$$

where $R90_t$ is the fluctuation intensity corresponding to a 5-minute measurement window *t* defined in Materials and Methods, $R90_{\text{1-day median}}$ and $R90_{\text{1-day 75-percentile}}$ are the median and 75-percentile of *R90* values during a 1-day period centered on *t*, respectively. $R90_{\text{12-hour median}}$ is the median of *R90* values during a 12-hour period centered on *t*. The upper thresholds of N_{CCN} are 1000, 1800, 2000, 2000, and 2000 cm^{-3} for supersaturation levels of 0.1%, 0.2%, 0.5%, 0.8%, and 1.0%, respectively. During shoreline-influencing periods, CCN fluctuation usually persists over several hours. In contrast, spikes in CCN concentration and fluctuation due to local anthropogenic pollution are generally short, isolated events (i.e., typically a few spikes over short periods during a day). These spikes can be easily identified using the first two criteria.

Given the strong impact of local pollution on $N_{>10}$, the periods influenced by local anthropogenic pollution were identified from the $N_{>10}$ value and its fluctuation. We derived a concentration spike flag at 1-minute resolution by examining the deviation of $N_{>10}$ at a particular time point compared with the median of $N_{>10}$ within a certain adjacent time period. This approach is similar to the one employed by Gallo (2020) (*83*). A spike flag value of 1 denotes $N_{>10}$ spike likely due to the influence of local emission, whereas a spike flag value of 0 indicates negligible impact from local pollution. Because nearshore SSA emission can also result in strong $N_{>10}$ fluctuations, we further investigate whether a spike flag of 1 is caused by nearshore SSA alone or influenced by anthropogenic pollution. During shoreline-influenced periods (according to the criterion 1 explained in Materials and Methods), if at least one minute of a 4-minute SMPS scan has spike flag of 1, we determine whether it is influenced by local anthropogenic pollution using the ratio of $N_{<40}$ to $N_{>250}$, based on the contrasting aerosol size distributions of anthropogenic pollution and

nearshore SSA. Here $N_{<40}$ and $N_{>250}$ denote the total number concentrations corresponding to particles smaller than 40 nm and larger than 250 nm, respectively. If the value of $N_{<40}/N_{>250}$ is below 13, we attribute the spike of $N_{>10}$ to nearshore SSA emission only (i.e., not influenced by anthropogenic pollution). Otherwise, aerosol size distributions measured by SMPS are flagged as influenced by pollution and excluded from the analysis. Outside the shoreline-influenced periods, all SMPS scans with at least one minute having spike flag of 1 are considered influenced by local pollution and removed.

The calculation of wind-wave Reynolds number

While surface wind speed is the primary driver for the wave breaking in open oceans, other factors such as wave fields and seawater properties also affect the breaking intensity and subsequent SSA emissions. Therefore, despite an overall strong correlation of whitecap fraction or SSA emission flux with wind speed, substantial scatter exists at a given wind speed level (*84*). It has been shown that the wind-wave Reynolds number ($\mathrm{Re}_{H_\mathrm{w}}$), which encapsulates the influences of wave height, wind history, friction velocity, and seawater viscosity, better explains the variation of SSA emission flux than wind speed alone (*39, 85*). Here we calculate the $\mathrm{Re}_{H_\mathrm{w}}$ value as an additional proxy of open-ocean SSA production, with the aim of better attributing the cause of observed strong aerosol population fluctuation and SSA enhancement. $\mathrm{Re}_{H_\mathrm{w}}$ is defined as:

$$\mathrm{Re}_{H_\mathrm{w}} = \frac{u_* H_{\mathrm{s,wind}}}{\nu_\mathrm{w}}$$

Here u_* is the friction velocity, which is given by $u_* = C_\mathrm{d}^{1/2} U_{10}$. C_d and U_{10} refer to the coefficient of drag with waves and 10-m wind speed, respectively. $H_{\mathrm{S,wind}}$ is the significant wave height of wind waves following the same approach in Ovadnevaite et al. (*39*). We did not use the significant wave height of total waves because it includes the contribution of nearshore wave breaking (*30*). ν_w is the kinematic viscosity of surface seawater, which is parameterized using sea surface temperature (SST) and salinity following the functions provided in Sharqawy et al. (*86*). To calculate the $\mathrm{Re}_{H_\mathrm{w}}$ value, we adopted C_d and H_S from the ERA-5. U_{10} is from in-situ measurement, SST is from MERRA-2, and the salinity is assumed as a constant of 0.035 kg kg^{-1}.

Spatiotemporal evolution of meteorological parameters associated with high-wave events in eastern North Atlantic

To further validate the hypothesis that the high-wave events near the ENA site are associated with cold air outbreaks and elucidate how synoptic processes affect ocean waves, we explored the spatiotemporal evolution of relevant meteorological parameters and ocean wave status on a large scale (90° W – 5° E, 15° N – 70° N). First, for each high-wave event taking place near the ENA site, the time point when H_S starts a rapid increase was set as the reference time. Then, the spatiotemporal changes of sea-level pressure, 10-m air temperature, U_{10}, and the H_S corresponding to wind waves ($H_{\mathrm{S,wind}}$), swell waves ($H_{\mathrm{S,swell}}$), and total waves (H_S) from 48 hours before to 48 hours after the reference time were examined. To remove the long-term variations, such as seasonal cycles, of the parameters, we derived their anomalies by subtracting 30-day centered running mean fields. During the one-year ACE-ENA campaign, a total of 44 such cases with a rapid H_S increase were identified. The mean spatiotemporal evolutions of the parameters are shown by the anomaly

fields averaged over all cases at each relative time point (fig. S8). The spatiotemporal evolution patterns of sea-level pressure and temperature clearly show the cold air outbreaks from North America or the Arctic and subsequent eastward movement to the eastern North Atlantic. High wind speed and $H_{S,wind}$ are induced by cold air outbreaks, and exhibit similar spatial patterns and temporal evolution. The area with the strongest surface wind speed is typically located to the north of the ENA site. After the passage of cold fronts, the wind speed and $H_{S,wind}$ decrease rapidly. However, the regional wave energy exhibits a slower dissipation rate and can propagate over thousands of kilometers in the form of long-period swell waves. Therefore, strong swell waves, which are mostly from the northwest, dominate the variation of H_S after the cold front passage, and the peak of $H_{S,swell}$ or H_S lags behind the peak of wind speed or $H_{S,wind}$. Similar analysis was also carried out for the evolutions of local meteorology, H_S, and aerosol properties measured at the ENA site. The results are presented in Fig. 3 and discussed in the main text.

The cold air outbreak in the North Atlantic is much more frequent in winter than in summer (*87*). The close association of strong waves with cold air outbreaks can explain the predominant northwest wave direction as well as the seasonal variation of H_S, which is higher in winter and lower in summer (fig. S9). It should be noted that other synoptic processes could also produce high waves, such as the extratropical cyclone storms (*88*). However, they are much less frequent than cold air outbreaks in the eastern North Atlantic throughout the year.

<u>Estimation of the contribution of nearshore SSA to total aerosol number concentration based on 1-minute data</u>

We applied the same fluctuation-based approach as described in the Materials and Methods to estimate the lower limit of the contribution of nearshore SSA to total aerosol number concentration during the TCAP campaign at Cape Cod. Because only 1-minute $N_{>10}$ data are available during the field campaign, we first estimated the difference between mean and minimum $N_{>10}$ during a 5-minute time window ($\Delta N_{>10}$) if the data had a 1-second time resolution. Here, based on the $N_{>10}$ data measured at the ENA site during the one-year ACE-ENA campaign, we find there is a close relationship between the $\Delta N_{>10}$ derived from 1-second raw data (Y) and the $\Delta N_{>10}$ (X) and minimum $N_{>10}$ (M) based on 1-minute averaged data: $\ln(Y) = a + b\ln(X) + c\ln^2(X) + d\ln(M)$ (fig. S18A). A similar strong relationship also exists for CCN concentration (fig. S18B), suggesting a general relationship related to the temporal variation of ambient aerosol number concentration. We reconstructed the time series of Y at Cape Cod from the measured 1-minute $N_{>10}$ data using the above relationships derived from the ENA data. The lower limit of the contribution of nearshore SSA to total aerosol number was then estimate from the reconstructed Y (i.e., 1 Hz $\Delta N_{>10}$).

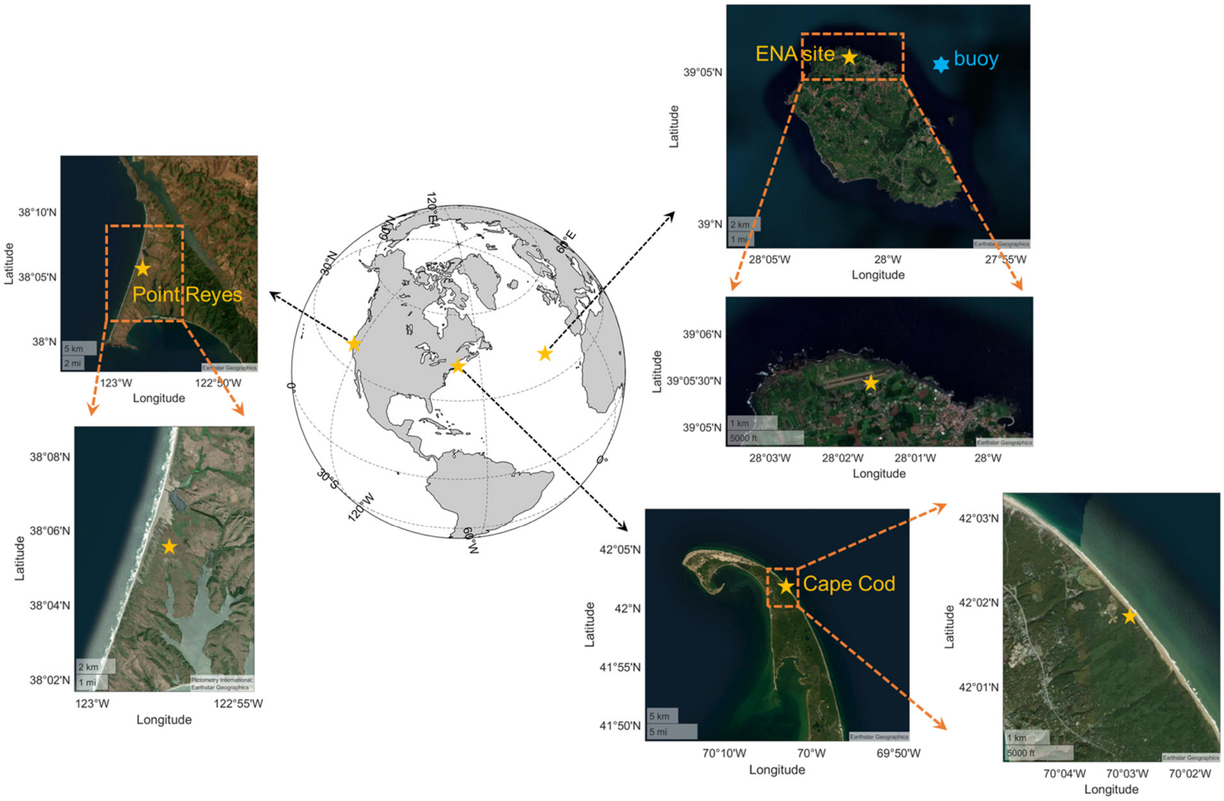

Fig. S1. The locations of observation sites. The closest distances of the ENA site and the sites at Cape Cod and Point Reyes to the shoreline are 470 m, 160 m, and 1160 m, respectively.

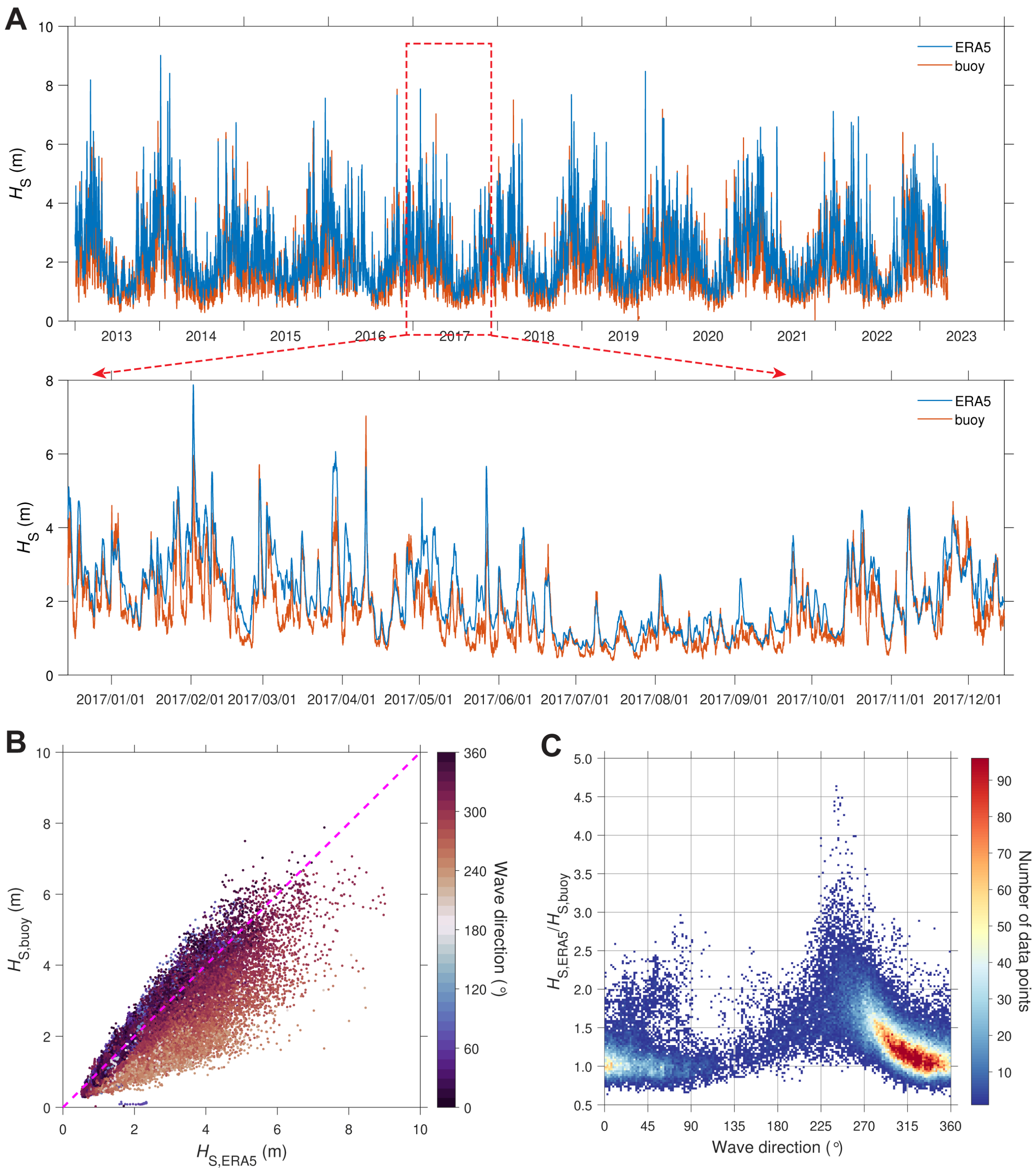

Fig. S2. Comparisons of the significant wave height near Graciosa Island from ERA5 and buoy measurements. (**A**) Time series of the significant wave height (H_S) from ERA5 and buoy observations from 2013 to 2023. (**B**) Scatter plot between the observed and ERA5 H_S colored by the wave direction. (**C**) The distribution of the ratio between ERA5 and observed H_S as a function of wave directions. The wave direction is from ERA5.

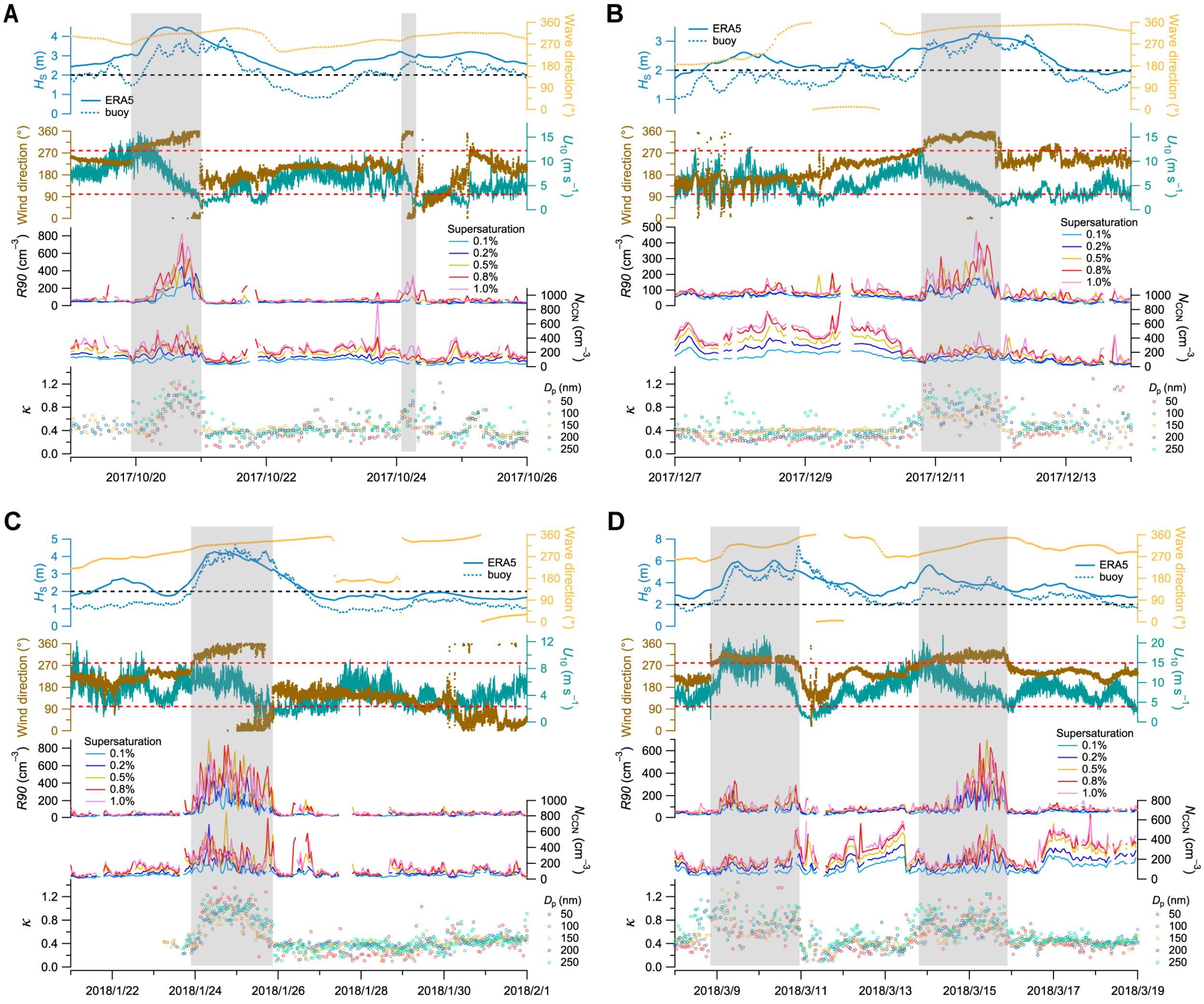

Fig. S3. Four additional examples showing the elevated CCN concentration fluctuation and aerosol hygroscopicity due to shoreline wave breaking.

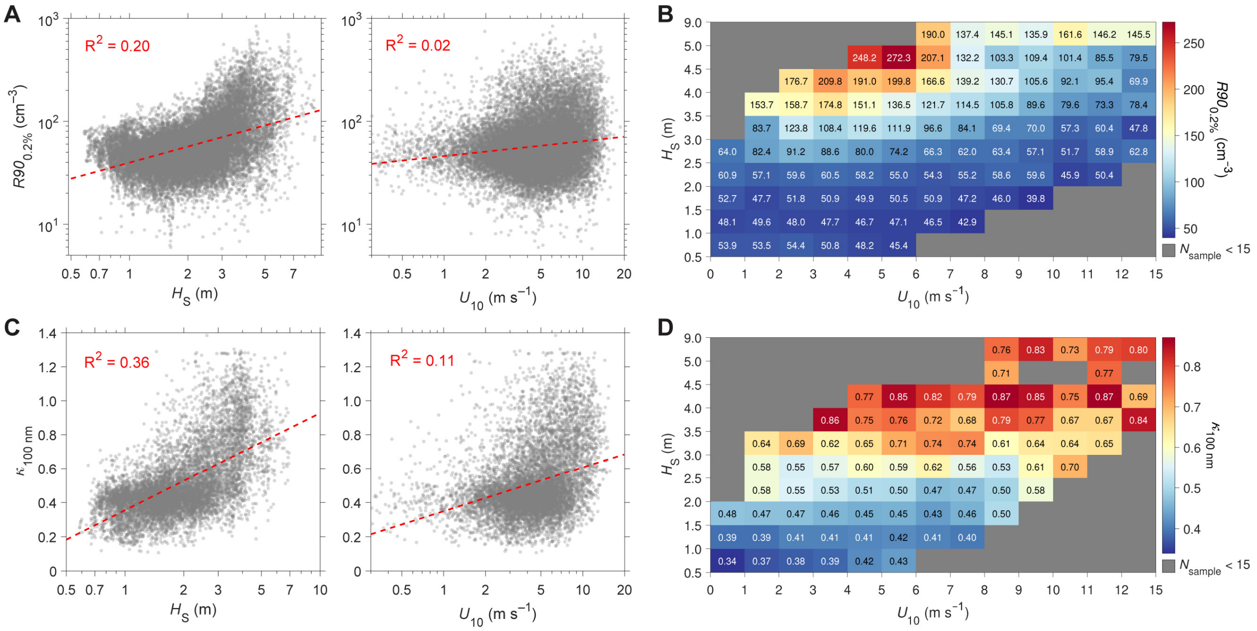

Fig. S4. The dependence of CCN fluctuation and aerosol hygroscopicity on H_S and U_{10}. (**A**) Correlations between CCN fluctuation (*R90*, supersaturation = 0.2%) and H_S and U_{10}. (**B**) Average *R90* values in different H_S and U_{10} ranges. (**C**) The same as panel A but for the κ values of particles with diameter of 100 nm ($\kappa_{100\ nm}$). (**D**) Average $\kappa_{100\ nm}$ values in different H_S and U_{10} ranges. All the data shown in this figure are with wind direction > 280° or < 100°. When wind direction is within these ranges, the ENA site is downwind of the shoreline wave-breaking area for vast majority of the time (time fraction = 96.0%), and the air mass travels a relatively short distance from the shoreline to the site (< 1.8 km). The H_S data in this figure are from ERA5.

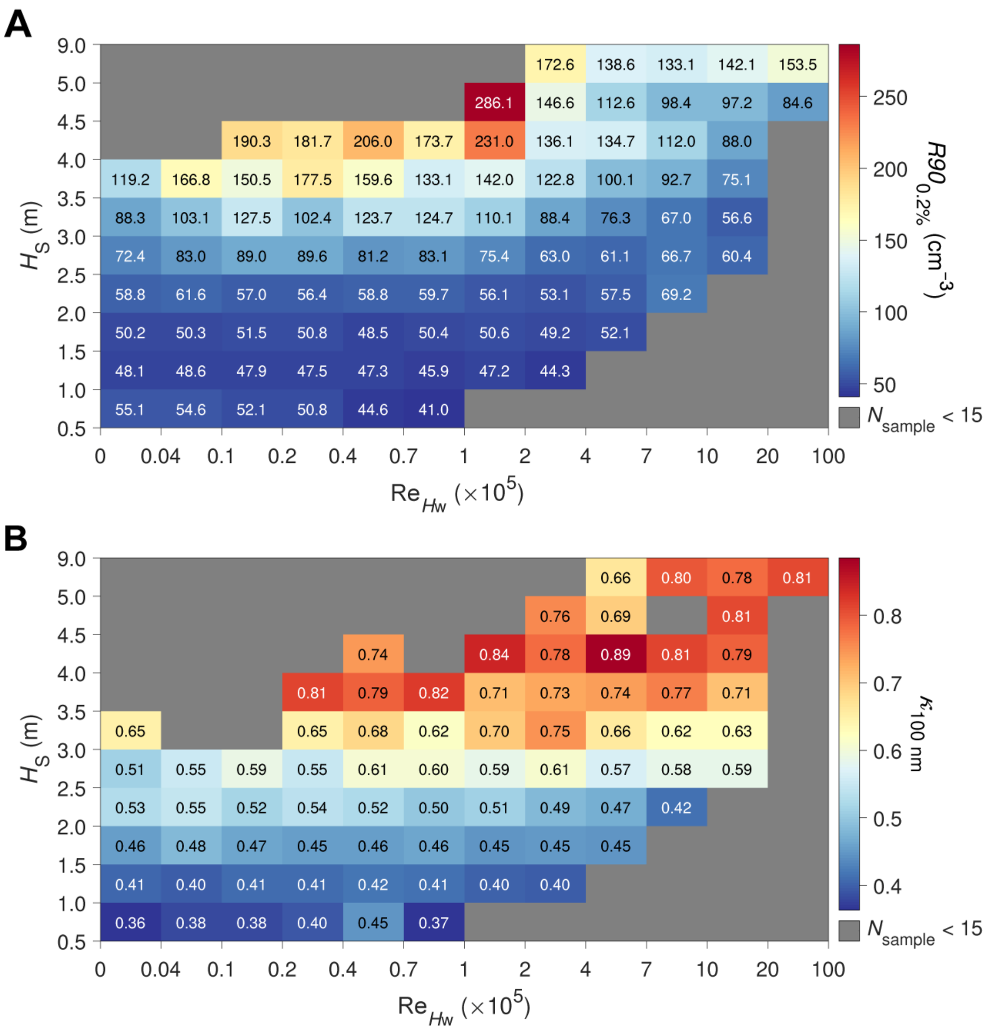

Fig. S5. The dependence of CCN fluctuation and aerosol hygroscopicity on H_S and wind-wave Reynolds number. (**A**) Average *R90* values in different H_S and wind-wave Reynolds number ($\mathbf{Re}_{H_w}$) ranges. (**B**) Average $\kappa_{100\ nm}$ values in different H_S and $\mathbf{Re}_{H_w}$ ranges. All the data shown in this figure are with wind direction > 280° or < 100°. The H_S data in this figure are from ERA5.

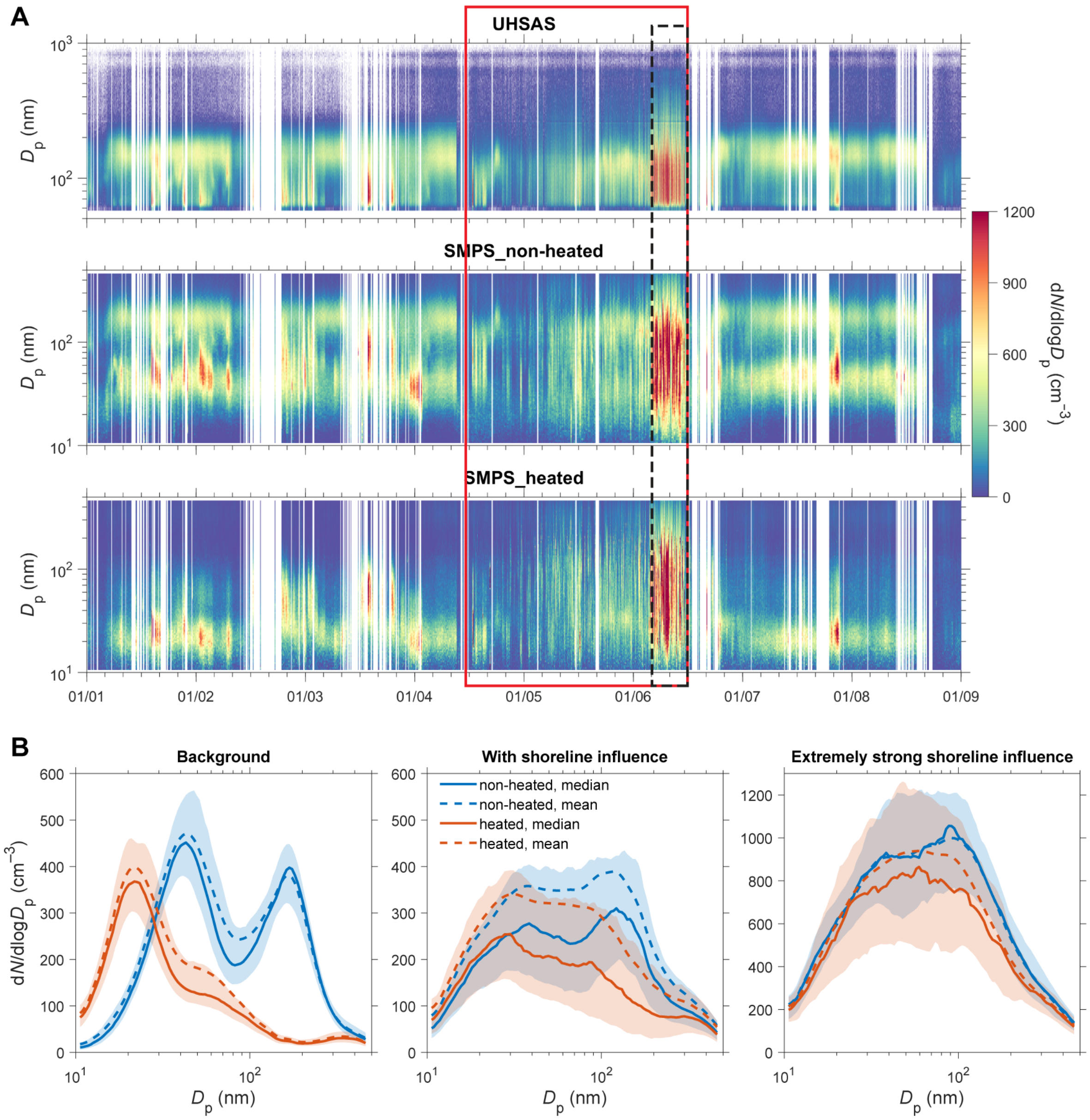

Fig. S6. Examples of aerosol number size distributions during background periods and periods with strong influence of nearshore SSA. (**A**) Aerosol number size distributions measured by UHSAS and SMPS from 1 January to 8 January 2018. Also shown are size distributions of aerosol samples heated to 300°C measured by the SMPS. The red box highlights the time period strongly influenced by nearshore SSA. The black dash box denotes the period with extremely strong influence of nearshore SSA, characterized by the strongest CCN fluctuation observed during the ACE-ENA campaign. During this period, the measured aerosols are dominated by nearshore SSA. (**B**) The comparison between aerosol size distributions during background periods, period strongly influenced by nearshore SSA, and period with extremely strong influence of nearshore SSA. The solid and dash lines represent the median and mean size distributions, respectively, while the shaded regions represent the ranges between the 25th and 75th percentiles.

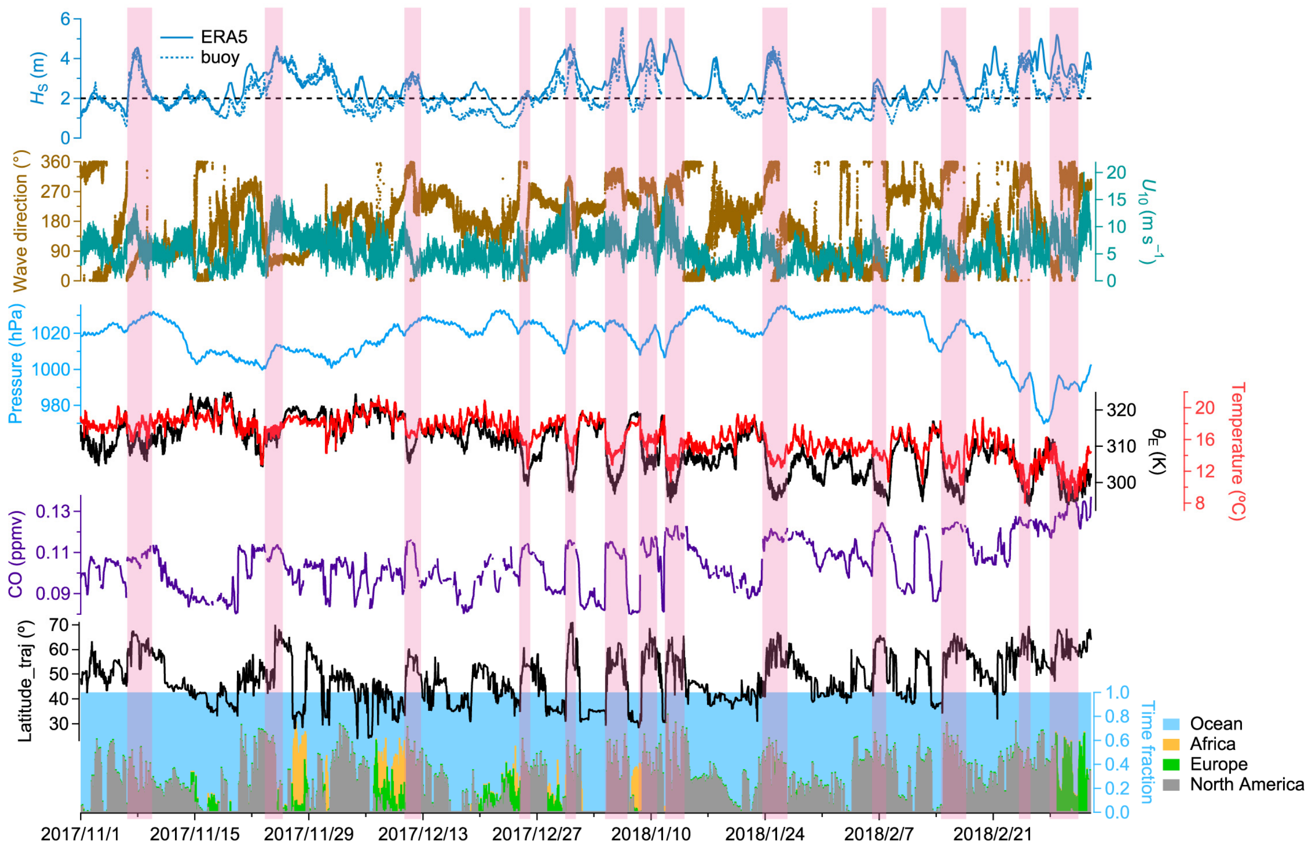

Fig. S7. Frequent high wave conditions near the ENA site induced by cold air outbreaks. This figure includes the time series of H_S, measured meteorological parameters (wind direction, 10-m wind speed U_{10}, pressure, equivalent potential temperature θ_E, and temperature), and CO concentration at the ENA site, and the time series of average latitude along 10-day air mass backward trajectories (Latitude_traj) and the time fractions of each trajectory spent over North America, Europe, Africa, and oceans. The backward trajectories of air masses arriving at the ENA site at an altitude of 200 m above the mean sea level were calculated using the HYbrid Single-Particle Lagrangian Integrated Trajectory (HYSPLIT) model (*89*). The periods shaded by light pink represent the high-wave events associated with the cold air outbreaks. For all but the last cold air outbreak event, air masses spent a notable fraction of time over North America during the 10 days prior to arriving at the ENA site.

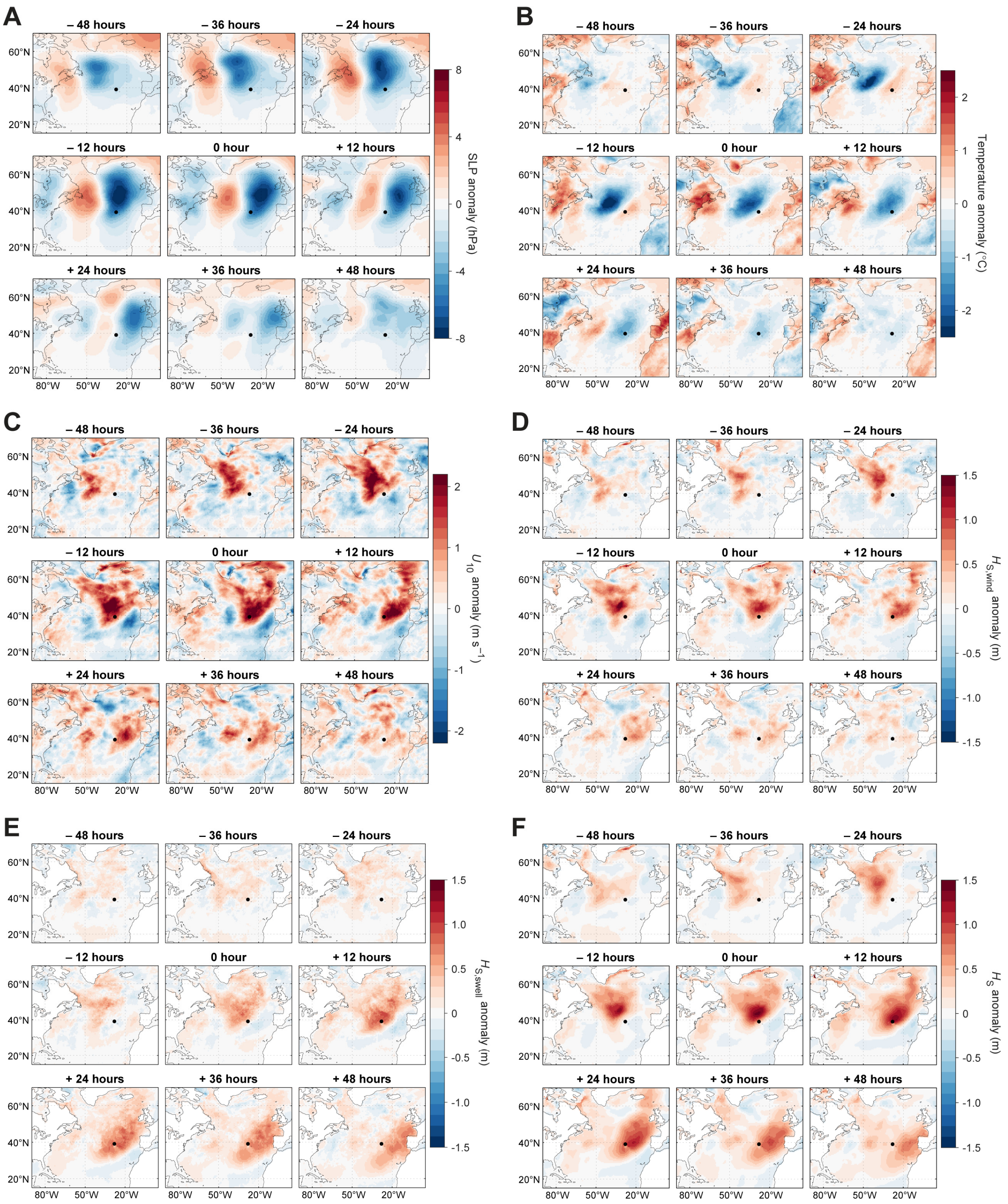

Fig. S8. The spatiotemporal evolution of cold air outbreaks and impacts on ocean wave status. (**A-C**) The average spatiotemporal evolution patterns of the anomalies of (A) sea-level pressure (SLP), (B) 10-m air temperature, and (C) 10-m wind speed (U_{10}) from 48 hours before to 48 hours after the onset of rapid increase in H_S at the ENA site. (**D-F**) The same as panels A-C but for (D) wind wave height ($H_{S,wind}$), (E) swell wave height ($H_{S,swell}$), and (F) total wave height (H_S) from ERA5. The black dot represents the location of the ENA site.

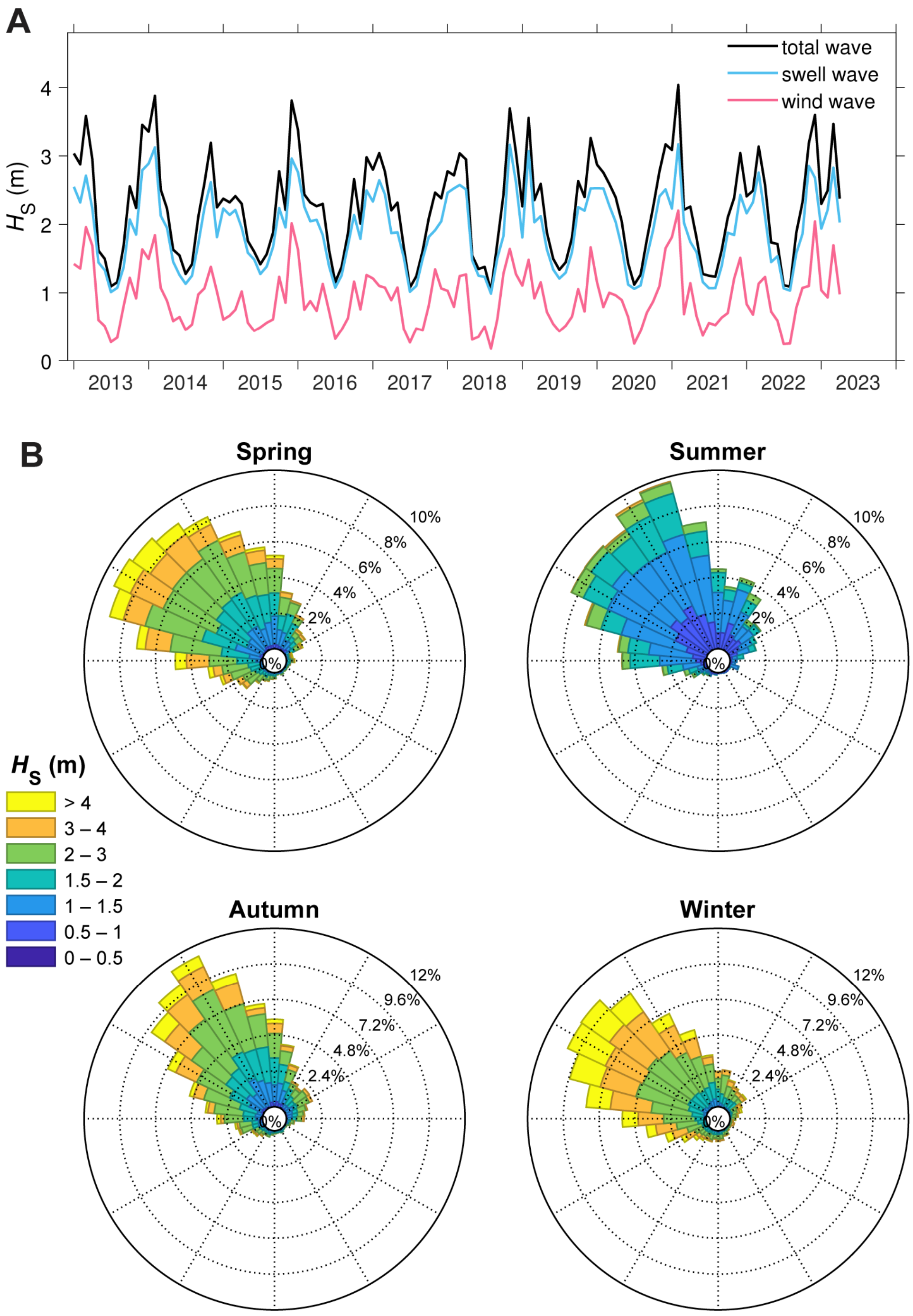

Fig. S9. Seasonal variations of H_S and wave direction distribution near the ENA site. (**A**) The monthly means of the H_S corresponding to total waves, swell waves, and wind waves from 2013 to 2023. (**B**) Wave direction roses for H_S (total waves) in different seasons. The wave data for deriving this figure are from ERA5.

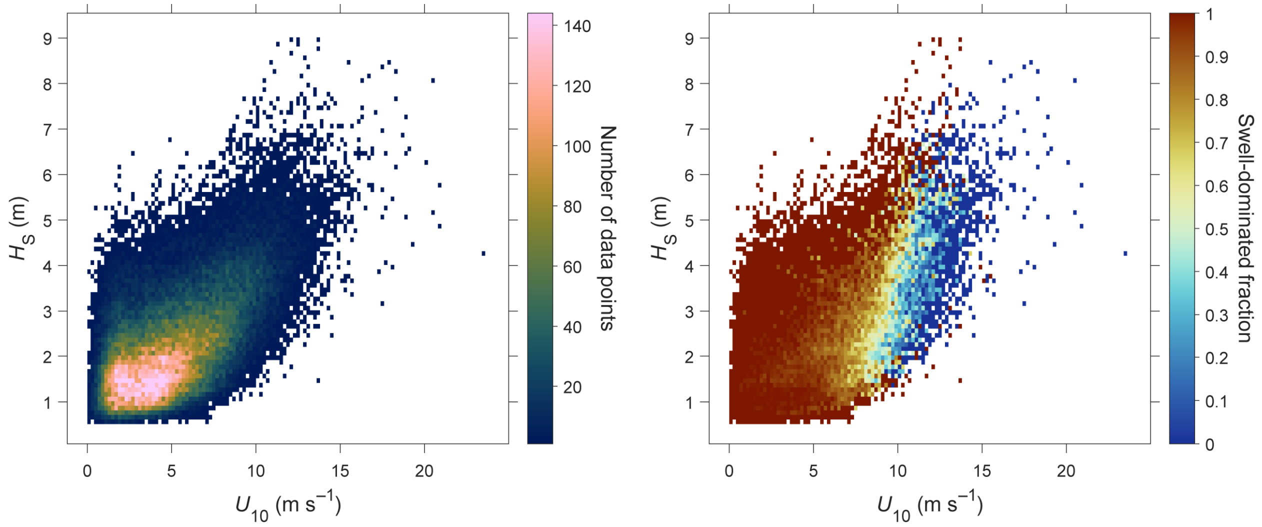

Fig. S10. The relationship between H_S and U_{10} at the ENA site. H_S and U_{10} are segregated with intervals of 0.1 m and 0.2 m s^{-1}, respectively. The number of data points in each 2-D H_S-U_{10} cell is shown in the plot on the left, and the corresponding time fraction with swell wave height exceeding wind wave height (swell-dominated fraction) is shown in the right plot. The wave data for deriving this figure are from ERA5.

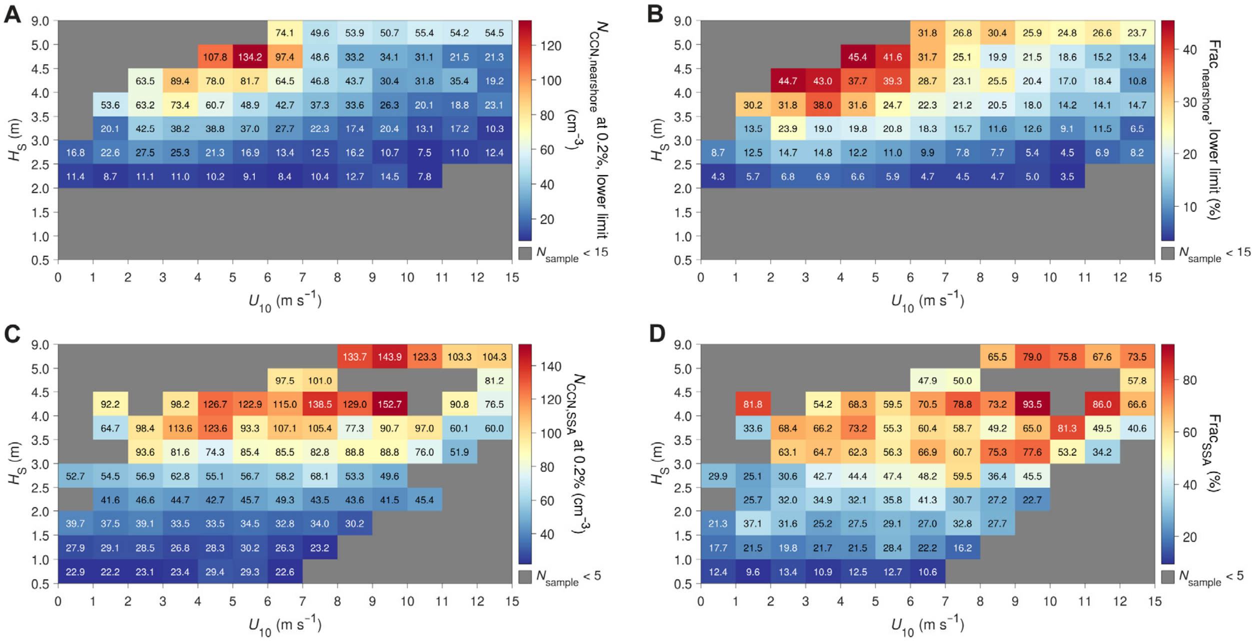

Fig. S11. The contribution of nearshore SSA to CCN in different H_S and U_{10} ranges. (**A** and **B**) Averages of the estimated (A) absolute and (B) relative contributions of nearshore SSA to CCN concentration in different H_S and U_{10} ranges. This estimation is based on the enhancement of CCN fluctuation, and it only represents the lower limit. (**C** and **D**) Averages of the (C) absolute and (D) relative contribution of SSA to CCN in different H_S and U_{10} ranges, derived from size-resolved aerosol hygroscopicity and aerosol size distribution. The H_S data in this figure are from ERA5.

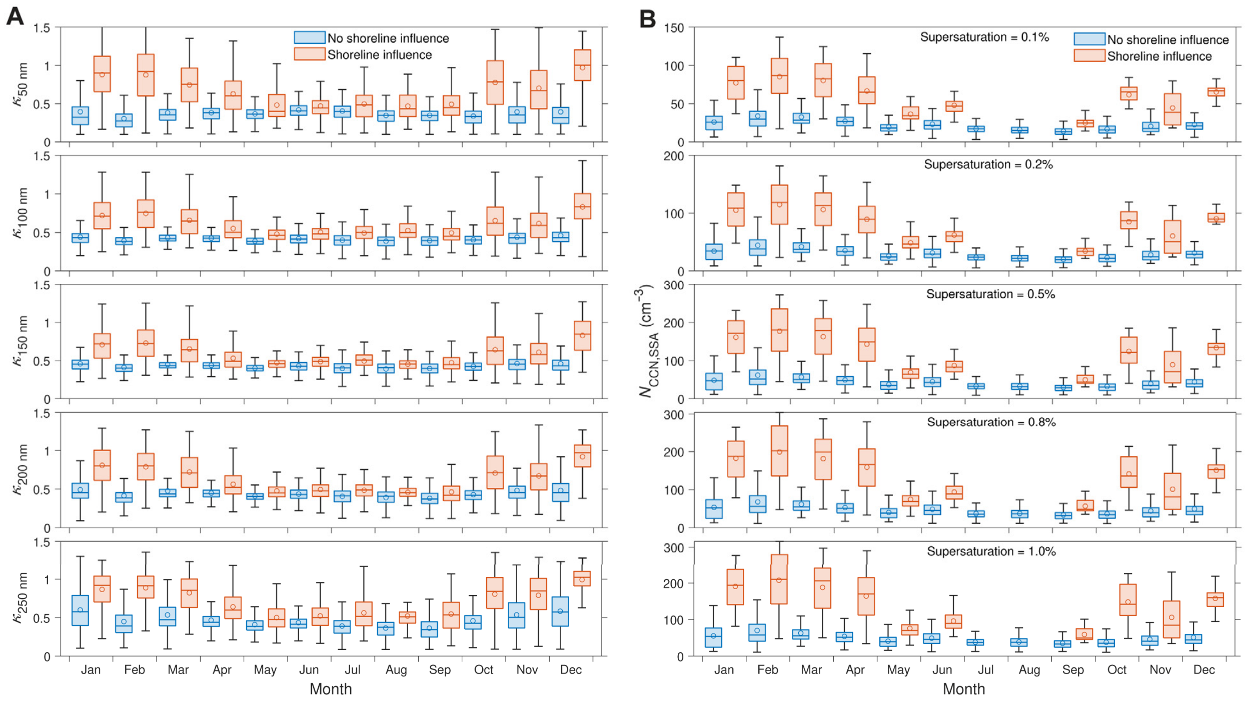

Fig. S12. Seasonal variations of (A) aerosol hygroscopicity parameter (*κ*) and (B) number concentration of SSA particles serving as CCN ($N_{\mathrm{CCN,SSA}}$) during background periods and shoreline-influencing periods. The boxes represent upper and lower quartiles, the horizontal lines and circles represent the median and mean values, respectively, and the whiskers represent the minimum and maximum values within 1.5 times the interquartile range (IQR) from the lower and upper quartiles. While multi-year data are available for hygroscopicity, only one-year aerosol size distribution data during ACE-ENA campaign are available for quantifying $N_{\mathrm{CCN,SSA}}$. As a result, the statistics of $N_{\mathrm{CCN,SSA}}$ during shoreline-influencing periods in July and August are not shown due to small numbers of data points (i.e., less than 10).

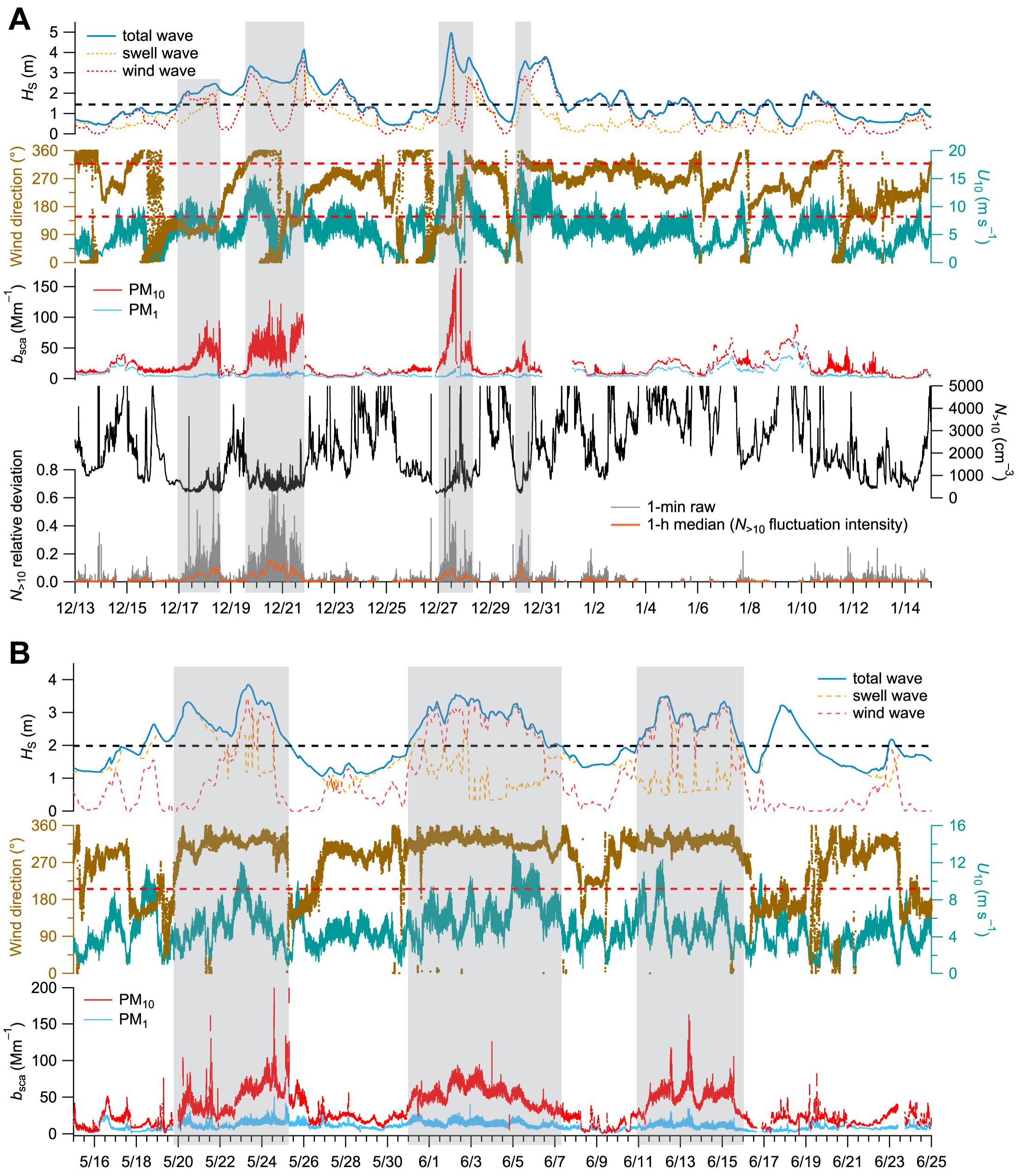

Fig. S13. Examples of the strong impact of nearshore SSA generation on aerosol populations at Cape Cod and Point Reyes measurement sites. (**A**) Time series of H_S for different wave components, wind direction, U_{10}, PM_1 and PM_{10} b_{sca}, $N_{>10}$, and relative deviation of $N_{>10}$ from 13 December 2012 to 14 January 2013 at Cape Cod. The definitions of the relative deviation of $N_{>10}$ and fluctuation intensity are detailed in Materials and Methods. (**B**) Time series of H_S for different wave components, wind direction, U_{10}, and PM_1 and PM_{10} b_{sca} from 15 May to 24 June 2009 at Point Reyes. For both panels, the shaded rectangular boxes indicate the time periods strongly influenced by shoreline wave breaking. The horizontal dashed lines represent the thresholds of H_S

and onshore wind direction sectors (Cape Cod: H_S > 1.5 m, wind direction > 320° or < 150°; Point Reyes: H_S > 2.0 m, wind direction > 200°). The wave data in this figure are from ERA5.

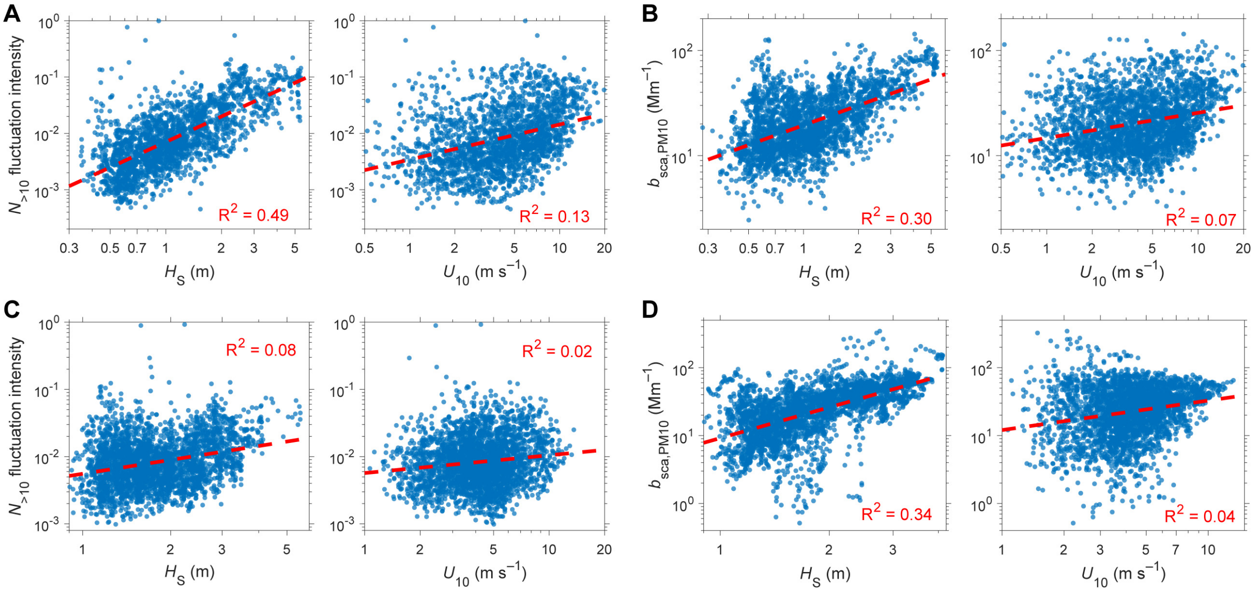

Fig. S14. Correlations of aerosol properties with H_S and U_{10}. (**A** and **B**) Correlations of (A) $N_{>10}$ fluctuation intensity and (B) PM_{10} scattering coefficient with H_S and U_{10} at Cape Cod. (**C** and **D**) The same as (A and B) but for Point Reyes. All correlations are under the condition of onshore winds, and the H_S data are from ERA5.

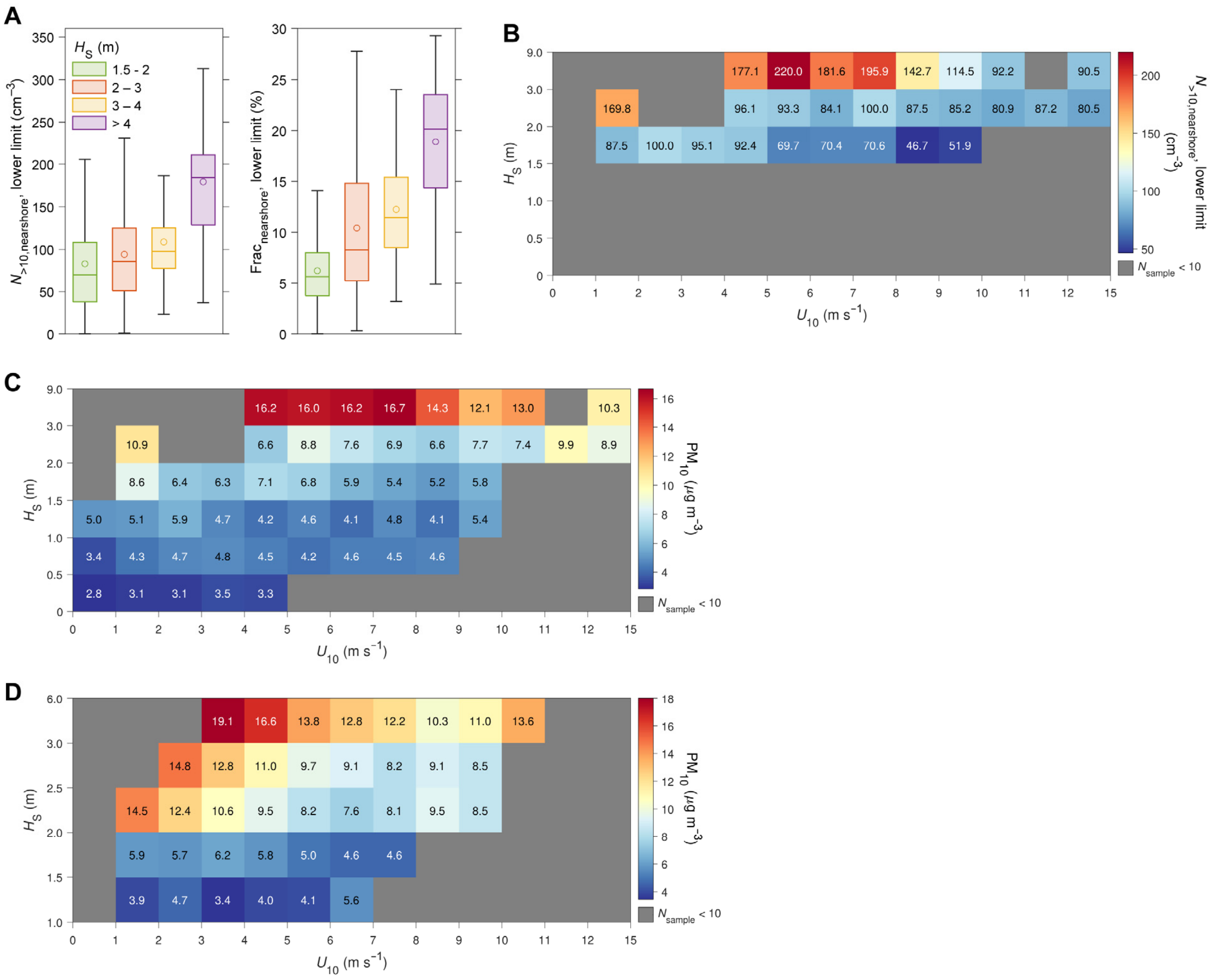

Fig. S15. The contributions of nearshore SSA to aerosol populations at Cape Cod and Point Reyes measurement sites. (**A**) The estimated absolute and relative contributions of nearshore SSA to observed $N_{>10}$ over Cape Cod grouped by different H_S ranges. The boxes represent upper and lower quartiles, the horizontal lines and circles represent the median and mean values, and the whiskers represent the minimum and maximum values within 1.5 times the interquartile range (IQR) from the lower and upper quartiles. (**B**) The average values of the estimated absolute contributions of nearshore SSA to observed $N_{>10}$ over Cape Cod in different H_S and U_{10} ranges. For panels A and B, the contribution was estimated from the enhancement of the fluctuation during the shoreline-influencing periods and only represents the lower limit. (**C**) Average PM$_{10}$ mass concentrations over Cape Cod in different H_S and U_{10} ranges under onshore winds conditions. (**D**) The same as panel C but for Point Reyes. All H_S data in this figure are from ERA5.

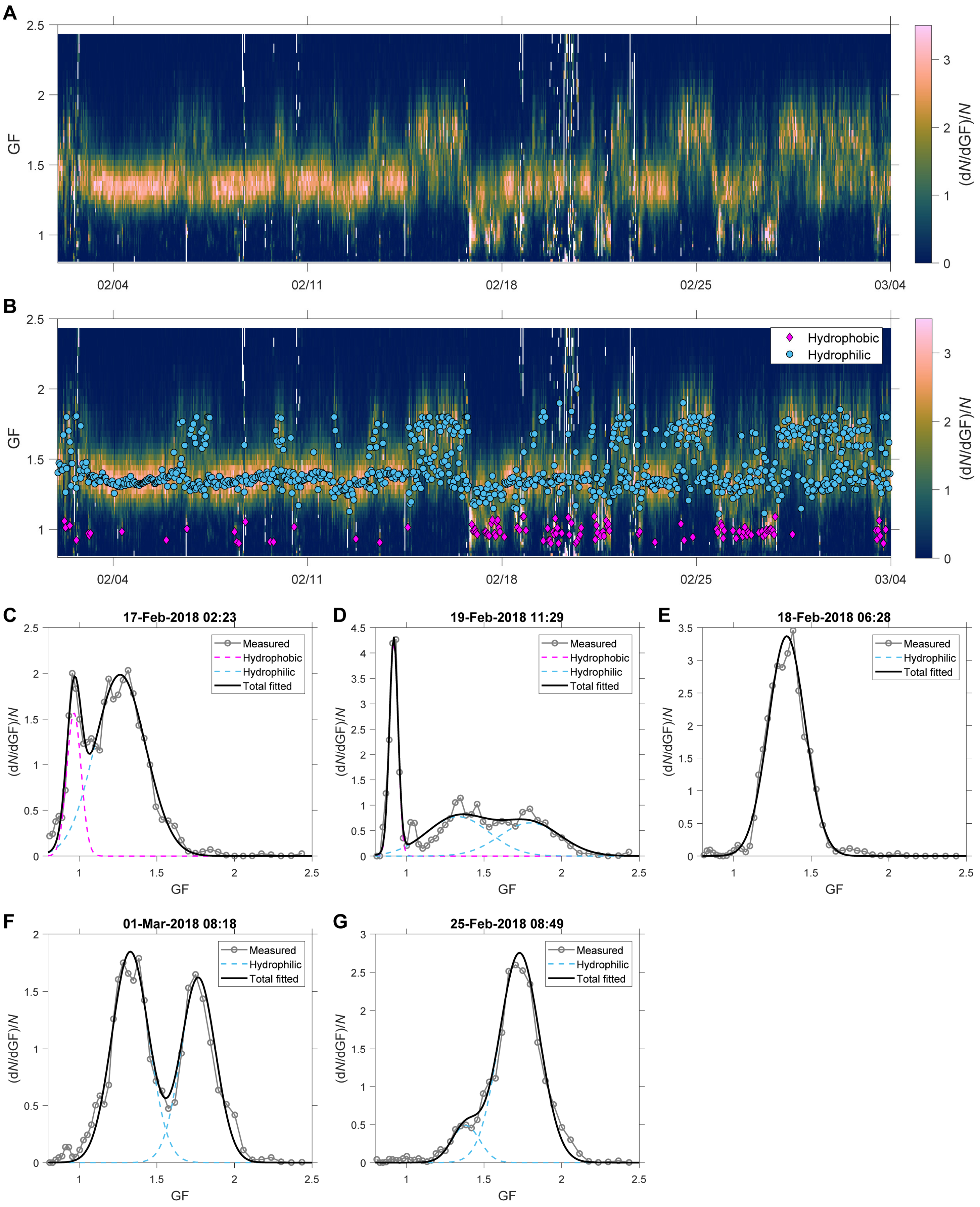

Fig. S16. Examples illustrating the mode fitting procedure for HTDMA-measured hygroscopic growth factor (GF). (**A**) Contour plot showing the time series of GF distribution for particles with a dry diameter of 100 nm from 2 February to 3 March 2018. (d*N*/dGF)/*N* is the number probability density function of GF. (**B**) The same as panel A but adding the average GF

of each fitted mode. (**C-G**) Examples of the mode fitting result for an individual GF distribution spectrum measured during a specific HTDMA scan. These examples show representative aerosol mixing states observed at the ENA site, including: (C) the mixture of hydrophobic and non-sea-salt hydrophilic modes; (D) the mixture of hydrophobic, non-sea-salt hydrophilic, and sea-salt modes; (E) a single non-sea-salt hydrophilic mode; (F) the mixture of non-sea-salt hydrophilic and sea-salt modes; (G) the same as (F) but dominated by sea-salt mode. The area of each mode represents its number fraction. The average κ of a hydrophilic mode was calculated based on the fitted mode GF. For the cases with multiple hydrophilic modes such as panels D, F, and G, the overall average κ was derived as the number-weighted average of these hydrophilic modes.

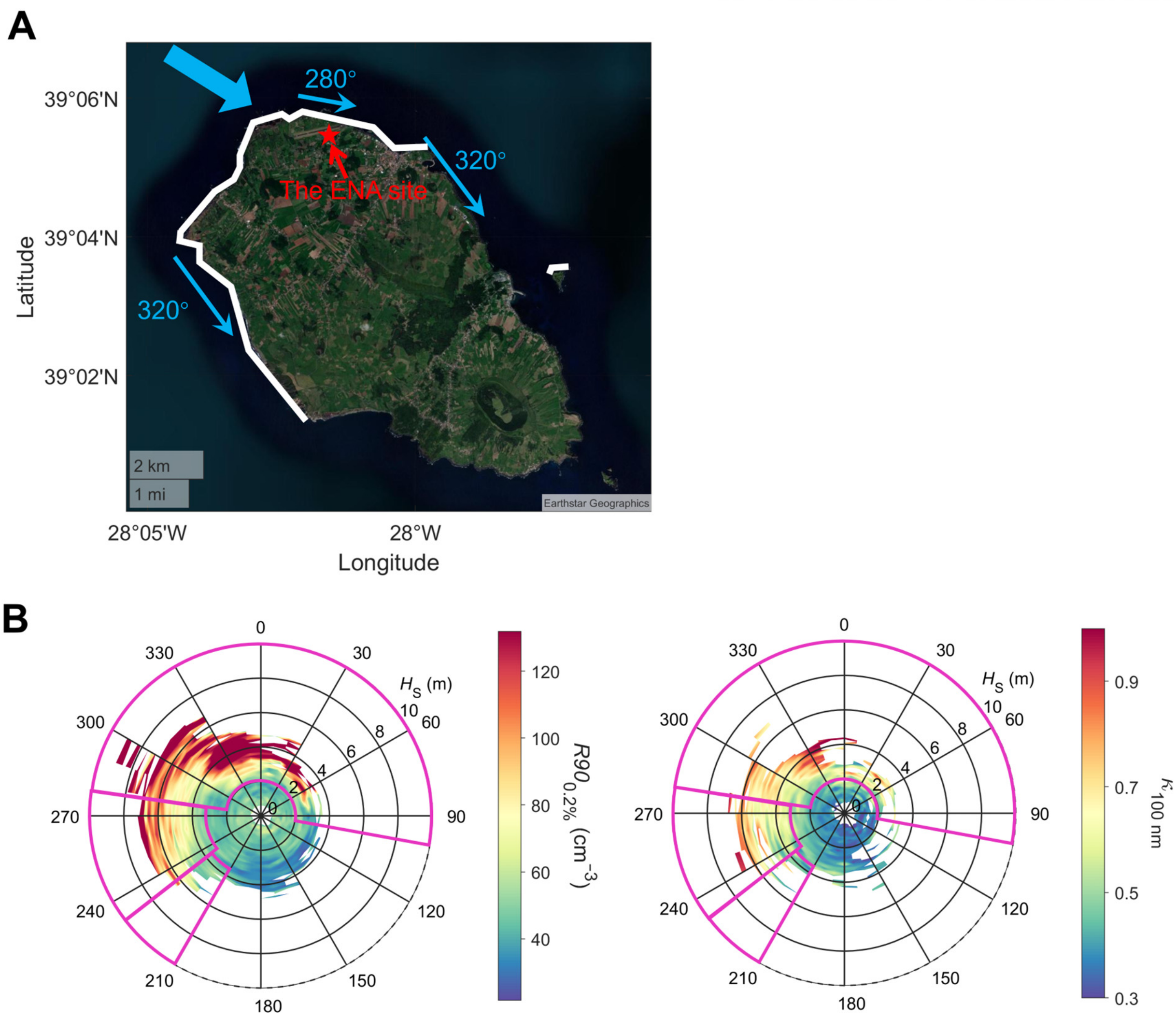

Fig. S17. An example illustrating how the criterion 1 for identifying the shoreline-influencing periods is established. (**A**) A schematic diagram showing where the wave breaking will occur along the shoreline (the thick white line) when waves come from the direction between 280° and 320°. (**B**) The dependence of *R90* (supersaturation = 0.2%) and κ (D_p = 100 nm) on wind direction and H_S when wave direction is between 280° and 320°. The regions enclosed by the pink lines represent the conditions satisfying the criterion 1, wherein H_S exceeds the respective threshold for each of the three wind direction sectors. The H_S data in this figure are from ERA5.

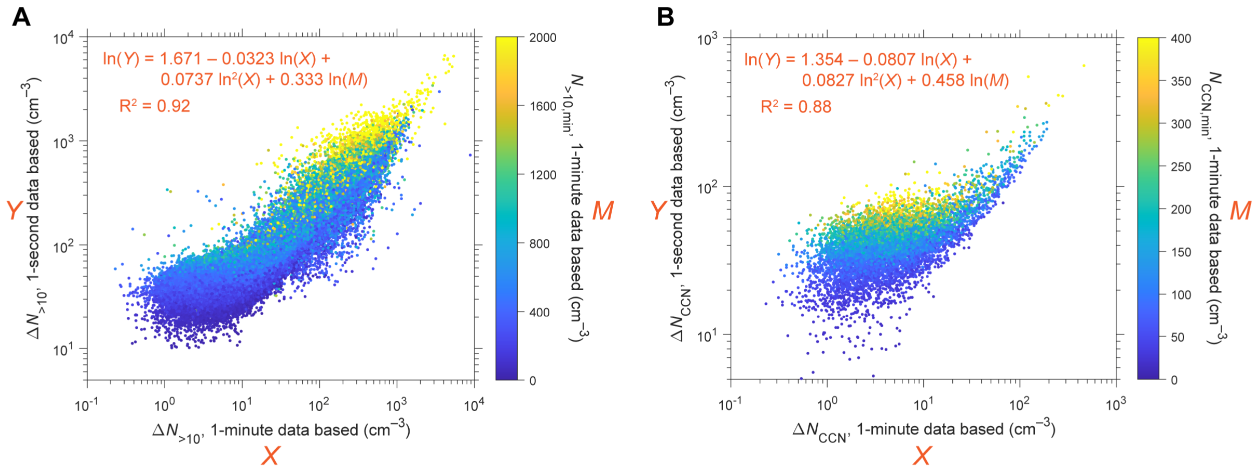

Fig. S18. (A) The dependence of the Δ$N_{>10}$ within a 5-minute time window derived from the 1-second raw data on the corresponding Δ$N_{>10}$ and $N_{>10,min}$ derived from the 1-minute averaged data within the same 5-minute window. Δ$N_{>10}$ is defined as the difference between mean and minimum aerosol total number concentrations within a 5-minute time window (i.e., $N_{>10,mean} - N_{>10,min}$). **(B) The same as panel A but for N_{CCN}.** The data are from the measurements at the ENA site. Dependence functions and corresponding R^2 values are also presented in the figure.

Table S1. Detailed information of the aerosol measurements used in this study.

Site	Measurement	Symbol	Unit	Instrument	Time resolution	Measurement period	Note
ENA site	Total number concentration of aerosol particles larger than 10 nm	$N_{>10}$	cm^{-3}	Condensation particle counter (CPC), Model 3772, TSI Inc.	1 second	October 2013 to April 2014<br>June 2015 to May 2023	
	Aerosol scattering coefficients	b_{sca}	Mm^{-1}	Integrating Nephelometer, Model 3563, TSI Inc.	5 seconds	October 2013 to May 2023	The 50% cut size of the Nephelometer inlet alternates between 1 μm and 10 μm
	CCN concentration at different supersaturation levels	N_{CCN}	cm^{-3}	Single-column cloud condensation nuclei counter (CCNC), Model CCN-100, Droplet Measurement Technologies	1 second	October 2013 to September 2015<br>June 2016 to June 2018<br>April 2019 to October 2020	supersaturation cycles though 0.1%, 0.2%, 0.5%, 0.8%, and 1.0% (changes every 10 min)
				Two-column CCNC, Model CCN-200, Droplet Measurement Technologies	1 second	January 2021 to April 2023	supersaturation cycles through 0.1%, 0.2%, 0.4%, 0.8%, and 1.0% in one column (changes every 10 min) and remains constant at 0.4% in the other column.
	Aerosol number size distribution (10 – 460 nm)	$dN/d\log D_p$	cm^{-3}	Scanning mobility particle sizer (SMPS), Model 3938, TSI Inc.	4 minutes for each scan	June 2017 to August 2018	Alternate between ambient samples and the samples after heated to 300 °C in a thermal denuder

	Aerosol number size distribution (60 nm – 1μm)	$dN/d\log D_p$	cm^{-3}	Ultra-High Sensitivity Aerosol Spectrometer (UHSAS), Droplet Measurement Technologies	10 seconds	June 2017 to August 2018	
	Aerosol hygroscopic growth factor and hygroscopicity parameter	GF, κ	unitless	Humidified Tandem Differential Mobility Analyzer (HTDMA), Model 3002, Brechtel Manufacturing Inc.	Before May 2019: 11.2 minutes for each scan, 56 minutes for one cycle<br>Since May 2019: 6.2 minutes for each scan, 31 minutes for one cycle	March 2017 to October 2018<br>May to December 2019<br>April 2022 to May 2023	Dry diameter cycles through 50, 100, 150, 200, and 250 nm
Cape Cod	Total number concentration of aerosol particles larger than 10 nm	$N_{>10}$	cm^{-3}	Condensation particle counter (CPC), Model 3772, TSI Inc.	1 minute	July 2012 to June 2013	
	Aerosol scattering coefficients	b_{sca}	Mm^{-1}	Integrating Nephelometer, Model 3563, TSI Inc.	1 minute	July 2012 to June 2013	
Point Reyes	Total number concentration of aerosol particles larger than 10 nm	$N_{>10}$	cm^{-3}	Condensation particle counter (CPC), Model 3772, TSI Inc.	1 minute	March to September 2005	
	Aerosol scattering coefficients	b_{sca}	Mm^{-1}	Integrating Nephelometer, Model 3563, TSI Inc.	1 minute	March to September 2005	

Table S2. Criterion 1 for identifying the shoreline-influencing periods at the ENA site.

Wave direction (°)	Wind direction (°)	H_S (m)
90 – 150	0 – 110	> 2
150 – 220	210 – 235	> 3.5
220 – 250	210 – 235	> 3.5
	235 – 280	> 3
250 – 280	210 – 235	> 3.5
	235 – 280	> 3
	280 – 360, 0 – 90	> 2
280 – 320	210 – 235	> 3.5
	235 – 280	> 3
	280 – 360, 0 – 100	> 2
320 – 30	235 – 280	> 3
	280 – 360, 0 – 110	> 2
30 – 90	280 – 360, 0 – 110	> 2

Table S3. Information of the 12 representative aerosol observation stations near the shoreline worldwide and corresponding criterion B in Fig. 5.

No.	Name	Lon (°)	Lat (°)	Altitude (m)	Distance to the shoreline (m)	Shoreline type	Criterion B		References
							Wave direction (°)	Wind direction (°)	
1	Point Reyes	-122.956	38.092	24	1160	beach	200 – 360, 0 – 15	200 – 360	(*90*)
2	La Jolla	-117.254	32.866	~10	0 (above a pier)	beach	230 – 360	200 – 360, 0 – 10	(*29*)
3	Cape Cod	-70.049	42.031	43	160	beach	310 – 360, 0 – 150	310 – 360, 0 – 150	(*91*)
4	Mace Head	-9.904	53.327	~20	~100	rocky	140 – 300	160 – 360	(*13*)
5	Tudor Hill	-64.879	32.264	51	52	rocky + beach	130 – 335	140 – 340	(*92*)
6	Cape Verde	-24.867	16.864	42	70	rocky	320 – 360, 0 – 180	250 – 360, 0 – 130	(*93*)
7	Ragged Point	-59.432	13.165	~30	46	rocky	305 – 360, 0 – 140	250 – 360, 0 – 150	(*94*)
8	Huaniao Island	122.673	30.861	~50	120	rocky	220 – 360, 0 – 120	220 – 360, 0 – 120	(*95*)
9	Cape Hedo	128.248	26.867	60	190	rocky	180 – 360, 0 – 130	180 – 360, 0 – 100	(*96*)
10	Cape Grim	144.689	-40.683	94	80	rocky	210 – 360	120 – 300	(*14*)
11	Baring Head	174.871	-41.408	~60	~300	beach	110 – 290	115 – 330	(*97*)
12	American Samoa	-170.565	-14.247	76	70	rocky	270 – 360, 0 – 180	260 – 360, 0 – 180	(*98*)

REFERENCES AND NOTES

1. J. H. Seinfeld, S. N. Pandis, *Atmospheric Chemistry and Physics: From Air Pollution to Climate Change* (John Wiley & Sons, 2016).

2. V. Ramanathan, P. J. Crutzen, J. Kiehl, D. Rosenfeld, Aerosols, climate, and the hydrological cycle. *Science* **294**, 2119–2124 (2001).

3. J. Wu, Evidence of sea spray produced by bursting bubbles. *Science* **212**, 324–326 (1981).

4. G. de Leeuw, E. L. Andreas, M. D. Anguelova, C. W. Fairall, E. R. Lewis, C. O'Dowd, M. Schulz, S. E. Schwartz, Production flux of sea spray aerosol. *Rev. Geophys.* **49**, doi.org/10.1029/2010RG000349 (2011).

5. C. Textor, M. Schulz, S. Guibert, S. Kinne, Y. Balkanski, S. Bauer, T. Berntsen, T. Berglen, O. Boucher, M. Chin, F. Dentener, T. Diehl, R. Easter, H. Feichter, D. Fillmore, S. Ghan, P. Ginoux, S. Gong, A. Grini, J. Hendricks, L. Horowitz, P. Huang, I. Isaksen, I. Iversen, S. Kloster, D. Koch, A. Kirkevåg, J. E. Kristjansson, M. Krol, A. Lauer, J. F. Lamarque, X. Liu, V. Montanaro, G. Myhre, J. Penner, G. Pitari, S. Reddy, Ø. Seland, P. Stier, T. Takemura, X. Tie, Analysis and quantification of the diversities of aerosol life cycles within AeroCom. *Atmos. Chem. Phys.* **6**, 1777–1813 (2006).

6. L. Deike, B. G. Reichl, F. Paulot, A mechanistic sea spray generation function based on the sea state and the physics of bubble bursting. *AGU Adv.* **3**, e2022AV000750 (2022).

7. L. E. Revell, N. E. Wotherspoon, O. J. Jones, Y. A. Bhatti, J. H. T. Williams, S. L. Mackie, J. P. Mulcahy, Atmosphere-ocean feedback from wind-driven sea spray aerosol production. *Geophys. Res. Lett.* **48**, e2020GL091900 (2021).

8. S. Liu, C. C. Liu, K. D. Froyd, G. P. Schill, D. M. Murphy, T. P. Bui, J. M. Dean-Day, B. Weinzierl, M. Dollner, G. S. Diskin, G. Chen, R. S. Gao, Sea spray aerosol concentration modulated by sea surface temperature. *Proc. Natl. Acad. Sci. U.S.A.* **118**, (2021).

9. A. Rap, C. E. Scott, D. V. Spracklen, N. Bellouin, P. M. Forster, K. S. Carslaw, A. Schmidt, G. Mann, Natural aerosol direct and indirect radiative effects. *Geophys. Res. Lett.* **40**, 3297–3301 (2013).

10. L. M. Russell, R. H. Moore, S. M. Burrows, P. K. Quinn, Ocean flux of salt, sulfate, and organic components to atmospheric aerosol. *Earth Sci. Rev.* **239**, 104364 (2023).

11. P. K. Quinn, D. J. Coffman, J. E. Johnson, L. M. Upchurch, T. S. Bates, Small fraction of marine cloud condensation nuclei made up of sea spray aerosol. *Nat. Geosci.* **10**, 674–679 (2017).

12. R. L. Modini, A. A. Frossard, L. Ahlm, L. M. Russell, C. E. Corrigan, G. C. Roberts, L. N. Hawkins, J. C. Schroder, A. K. Bertram, R. Zhao, A. K. Y. Lee, J. P. D. Abbatt, J. Lin, A. Nenes, Z. Wang, A. Wonaschütz, A. Sorooshian, K. J. Noone, H. Jonsson, J. H. Seinfeld, D. Toom-Sauntry, A. M. Macdonald, W. R. Leaitch, Primary marine aerosol-cloud interactions off the coast of California. *J. Geophys. Res. Atmos.* **120**, 4282–4303 (2015).

13. W. Xu, J. Ovadnevaite, K. N. Fossum, C. Lin, R.-J. Huang, D. Ceburnis, C. O'Dowd, Sea spray as an obscured source for marine cloud nuclei. *Nat. Geosci.* **15**, 282–286 (2022).

14. L. T. Cravigan, Z. Ristovski, R. L. Modini, M. D. Keywood, J. L. Gras, Observation of sea-salt fraction in sub-100 nm diameter particles at Cape Grim. *J. Geophys. Res. Atmos.* **120**, 1848–1864 (2015).

15. W. Xu, J. Ovadnevaite, K. N. Fossum, C. Lin, R. J. Huang, C. O'Dowd, D. Ceburnis, Seasonal trends of aerosol hygroscopicity and mixing state in clean marine and polluted continental air masses over the Northeast Atlantic. *J. Geophys. Res. Atmos.* **126**, e2020JD033851 (2021).

16. X. Hao, L. Shen, Wind–wave coupling study using LES of wind and phase-resolved simulation of nonlinear waves. *J. Fluid Mech.* **874**, 391–425 (2019).

17. K. Hasselmann, T. P. Barnett, E. Bouws, H. Carlson, D. E. Cartwright, K. Enke, J. Ewing, A. Gienapp, D. Hasselmann, P. Kruseman, "Measurements of wind-wave growth and swell decay during the Joint North Sea Wave Project (JONSWAP)" (Ergaenzungsheft zur Deutschen Hydrographischen Zeitschrift, Reihe A, Deutsches Hydrographisches Institut, 1973).

18. G. J. Komen, L. Cavaleri, M. Donelan, K. Hasselmann, S. Hasselmann, P. Janssen, *Dynamics and Modelling of Ocean Waves* (Cambridge Univ. Press, 1996).

19. X. Hao, L. Shen, Large-eddy simulation of gusty wind turbulence over a travelling wave. *J. Fluid Mech.* **946**, A8 (2022).

20. F. Saïd, Z. Jelenak, P. S. Chang, M. D. Anguelova, M. H. Bettenhausen, in *IGARSS 2024 - 2024 IEEE International Geoscience and Remote Sensing Symposium* (IEEE, 2024), pp. 5823–5826.

21. Y. Sugihara, H. Tsumori, T. Ohga, H. Yoshioka, S. Serizawa, Variation of whitecap coverage with wave-field conditions. *J. Mar. Syst.* **66**, 47–60 (2007).

22. A. Semedo, K. Sušelj, A. Rutgersson, A. Sterl, A global view on the wind sea and swell climate and variability from ERA-40. *J. Climate* **24**, 1461–1479 (2011).

23. H. Jiang, Z. Yang, A revisit of global wind-sea and swell climate and variability using multiplatform altimeters. *Remote Sens. Environ.* **271**, 112922 (2022).

24. G. Tedeschi, A. M. J. van Eijk, J. Piazzola, J. T. Kusmierczyk-Michulec, Influence of the surf zone on the marine aerosol concentration in a coastal area. *Bound.-Lay. Meteorol.* **163**, 327–350 (2017).

25. E. L. Andreas, Sea spray generation at a rocky shoreline. *J. Appl. Meteorol. Climatol.* **55**, 2037–2052 (2016).

26. X. Hao, Quantifying bioluminescent light intensity in breaking waves using numerical simulations. *Geophys. Res. Lett.* **51**, e2024GL110884 (2024).

27. G. Rojas, M. R. Loewen, Void fraction measurements beneath plunging and spilling breaking waves. *J. Geophys. Res. Oceans* **115**, C08001 (2010).

28. G. de Leeuw, F. P. Neele, M. Hill, M. H. Smith, E. Vignati, Production of sea spray aerosol in the surf zone. *J. Geophys. Res. Atmos.* **105**, 29397–29409 (2000).

29. A. M. J. van Eijk, J. T. Kusmierczyk-Michulec, M. J. Francius, G. Tedeschi, J. Piazzola, D. L. Merritt, J. D. Fontana, Sea-spray aerosol particles generated in the surf zone. *J. Geophys. Res.* **116**, D19210 (2011).

30. M. Yang, S. J. Norris, T. G. Bell, I. M. Brooks, Sea spray fluxes from the southwest coast of the United Kingdom – Dependence on wind speed and wave height. *Atmos. Chem. Phys.* **19**, 15271–15284 (2019).

31. T. Petelski, M. Chomka, Sea salt emission from the coastal zone. *Oceanologia* **42**, 399–410 (2000).

32. T. Zielinski, Aerosol masses and mass gradients in the marine boundary layer over the breaker zone. *Oceanologia* **39**, 201–209 (1997).

33. G. J. Kunz, G. de Leeuw, E. Becker, C. D. O'Dowd, Lidar observations of atmospheric boundary layer structure and sea spray aerosol plumes generation and transport at Mace Head, Ireland (PARFORCE experiment). *J. Geophys. Res.* **107**, 8106 (2002).

34. W. P. Hopper, L. U. Martin, Scanning lidar measurements of surf-zone aerosol generation. *Opt. Eng.* **38**, 250–255 (1999).

35. J. M. Brady, M. D. Stokes, J. Bonnardel, T. H. Bertram, Characterization of a quadrotor unmanned aircraft system for aerosol-particle-concentration measurements. *Environ. Sci. Technol.* **50**, 1376–1383 (2016).

36. K. L. Ackerman, A. D. Nugent, C. Taing, Mechanisms controlling giant sea salt aerosol size distributions along a tropical orographic coastline. *Atmos. Chem. Phys.* **23**, 13735–13753 (2023).

37. M. Smith, P. Park, I. Consterdine, Marine aerosol concentrations and estimated fluxes over the sea. *Q. J. Roy. Meteorol. Soc.* **119**, 809–824 (1993).

38. J. Ovadnevaite, D. Ceburnis, M. Canagaratna, H. Berresheim, J. Bialek, G. Martucci, D. R. Worsnop, C. O'Dowd, On the effect of wind speed on submicron sea salt mass concentrations and source fluxes. *J. Geophys. Res. Atmos.* **117**, D16201 (2012).

39. J. Ovadnevaite, A. Manders, G. de Leeuw, D. Ceburnis, C. Monahan, A. I. Partanen, H. Korhonen, C. D. O'Dowd, A sea spray aerosol flux parameterization encapsulating wave state. *Atmos. Chem. Phys.* **14**, 1837–1852 (2014).

40. S. L. Gong, L. A. Barrie, J. M. Prospero, D. L. Savoie, G. P. Ayers, J.-P. Blanchet, L. Spacek, Modeling sea-salt aerosols in the atmosphere: 2. Atmospheric concentrations and fluxes. *J. Geophys. Res. Atmos.* **102**, 3819–3830 (1997).

41. L. Jaeglé, P. K. Quinn, T. S. Bates, B. Alexander, J. T. Lin, Global distribution of sea salt aerosols: New constraints from in situ and remote sensing observations. *Atmos. Chem. Phys.* **11**, 3137–3157 (2011).

42. H. Grythe, J. Ström, R. Krejci, P. Quinn, A. Stohl, A review of sea-spray aerosol source functions using a large global set of sea salt aerosol concentration measurements. *Atmos. Chem. Phys.* **14**, 1277–1297 (2014).

43. P. Jiménez-Guerrero, O. Jorba, M. T. Pay, J. P. Montávez, S. Jerez, J. J. Gómez-Navarro, J. M. Baldasano, Comparison of two different sea-salt aerosol schemes as implemented in air quality models applied to the Mediterranean Basin. *Atmos. Chem. Phys.* **11**, 4833–4850 (2011).

44. E. Vignati, G. de Leeuw, R. Berkowicz, Modeling coastal aerosol transport and effects of surf-produced aerosols on processes in the marine atmospheric boundary layer. *J. Geophys. Res. Atmos.* **106**, 20225–20238 (2001).

45. E. Ternon, M. L. Carter, L. Cancelada, R. H. Lampe, A. E. Allen, C. R. Anderson, K. A. Prather, W. H. Gerwick, Yessotoxin production and aerosolization during the unprecedented red tide of 2020 in southern California. *Elementa-Sci. Anthrop.* **11**, (2023).

46. M. A. Pendergraft, P. Belda-Ferre, D. Petras, C. K. Morris, B. A. Mitts, A. T. Aron, M. Bryant, T. Schwartz, G. Ackermann, G. Humphrey, E. Kaandorp, P. C. Dorrestein, R. Knight, K. A. Prather, Bacterial and chemical evidence of coastal water pollution from the Tijuana River in sea spray aerosol. *Environ. Sci. Technol.* **57**, 4071–4081 (2023).

47. E. Van Acker, S. Huysman, M. De Rijcke, J. Asselman, K. A. C. De Schamphelaere, L. Vanhaecke, C. R. Janssen, Phycotoxin-enriched sea spray aerosols: Methods, mechanisms, and human exposure. *Environ. Sci. Technol.* **55**, 6184–6196 (2021).

48. B. Sha, J. H. Johansson, M. E. Salter, S. M. Blichner, I. T. Cousins, Constraining global transport of perfluoroalkyl acids on sea spray aerosol using field measurements. *Sci. Adv.* **10**, eadl1026 (2024).

49. V. P. Ghate, T. Surleta, L. Magaritz-Ronen, S. Raveh-Rubin, F. Gallo, A. G. Carlton, E. B. Azevedo, Drivers of cloud condensation nuclei in the Eastern North Atlantic as observed at the ARM site. *J. Geophys. Res. Atmos.* **128**, e2023JD038636 (2023).

50. A. D. Clarke, S. R. Owens, J. Zhou, An ultrafine sea-salt flux from breaking waves: Implications for cloud condensation nuclei in the remote marine atmosphere. *J. Geophys. Res.* **111**, D06202 (2006).

51. W. A. Hoppel, G. M. Frick, R. E. Larson, Effect of nonprecipitating clouds on the aerosol size distribution in the marine boundary layer. *Geophys. Res. Lett.* **13**, 125–128 (1986).

52. M. Chomka, T. Petelski, Modelling the sea aerosol emission in the coastal zone. *Oceanologia* **39**, 211–215 (1997).

53. B. G. Henderson, P. Chylek, W. M. Porch, M. K. Dubey, Satellite remote sensing of aerosols generated by the Island of Nauru. *J. Geophys. Res. Atmos.* **111**, doi.org/10.1029/2005JD006850 (2006).

54. S. Barthel, I. Tegen, R. Wolke, Do new sea spray aerosol source functions improve the results of a regional aerosol model? *Atmos. Environ.* **198**, 265–278 (2019).

55. Y. Chen, Y. Cheng, N. Ma, R. Wolke, S. Nordmann, S. Schüttauf, L. Ran, B. Wehner, W. Birmili, H. A. C. D. van der Gon, Q. Mu, S. Barthel, G. Spindler, B. Stieger, K. Müller, G.-J. Zheng, U. Pöschl, H. Su, A. Wiedensohler, Sea salt emission, transport and influence on size-segregated nitrate simulation: A case study in northwestern Europe by WRF-Chem. *Atmos. Chem. Phys.* **16**, 12081–12097 (2016).

56. D. Neumann, V. Matthias, J. Bieser, A. Aulinger, M. Quante, A comparison of sea salt emission parameterizations in northwestern Europe using a chemistry transport model setup. *Atmos. Chem. Phys.* **16**, 9905–9933 (2016).

57. B. Gantt, J. T. Kelly, J. O. Bash, Updating sea spray aerosol emissions in the Community Multiscale Air Quality (CMAQ) model version 5.0.2. *Geosci. Model Dev.* **8**, 3733–3746 (2015).

58. E. Athanasopoulou, M. Tombrou, S. N. Pandis, A. G. Russell, The role of sea-salt emissions and heterogeneous chemistry in the air quality of polluted coastal areas. *Atmos. Chem. Phys.* **8**, 5755–5769 (2008).

59. E. M. Knipping, D. Dabdub, Impact of chlorine emissions from sea-salt aerosol on coastal urban ozone. *Environ. Sci. Technol.* **37**, 275–284 (2003).

60. C. Harb, N. Pokhrel, H. Foroutan, Quantification of the emission of atmospheric microplastics and nanoplastics via sea spray. *Environ. Sci. Technol. Lett.* **10**, 513–519 (2023).

61. A. H. Woodcock, Note concerning human respiratory irritation associated with high concentrations of plankton and mass mortality of marine organisms. *J. Mar. Res.* **7**, 56–62 (1948).

62. L. E. Fleming, L. C. Backer, D. G. Baden, Overview of aerosolized Florida red tide toxins: Exposures and effects. *Environ. Health Perspect.* **113**, 618–620 (2005).

63. Y. S. Cheng, T. A. Villareal, Y. Zhou, J. Gao, R. Pierce, J. Naar, D. G. Baden, Characterization of red tide aerosol on the Texas coast. *Harmful Algae* **4**, 87–94 (2005).

64. M. Shechner, E. Tas, Ozone formation induced by the impact of reactive bromine and iodine species on photochemistry in a polluted marine environment. *Environ. Sci. Technol.* **51**, 14030–14037 (2017).

65. D. Lowenthal, N. Kumar, Light scattering from sea-salt aerosols at Interagency Monitoring of Protected Visual Environments (IMPROVE) sites. *J. Air Waste Manage. Assoc.* **56**, 636–642 (2006).

66. M. Casas-Prat, M. A. Hemer, G. Dodet, J. Morim, X. L. Wang, N. Mori, I. Young, L. Erikson, B. Kamranzad, P. Kumar, M. Menéndez, Y. Feng, Wind-wave climate changes and their impacts. *Nat. Rev. Earth Environ.* **5**, 23–42 (2024).

67. J. Uin, A. C. Aiken, M. K. Dubey, C. Kuang, M. Pekour, C. Salwen, A. J. Sedlacek, G. Senum, S. Smith, J. Wang, T. B. Watson, S. R. Springston, Atmospheric Radiation Measurement (ARM) Aerosol Observing Systems (AOS) for surface-based in situ atmospheric aerosol and trace gas measurements. *J. Atmos. Oceanic Tech.* **36**, 2429–2447 (2019).

68. J. Wang, R. Wood, M. P. Jensen, J. C. Chiu, Y. Liu, K. Lamer, N. Desai, S. E. Giangrande, D. A. Knopf, P. Kollias, A. Laskin, X. Liu, C. Lu, D. Mechem, F. Mei, M. Starzec, J. Tomlinson, Y. Wang, S. S. Yum, G. Zheng, A. C. Aiken, E. B. Azevedo, Y. Blanchard, S. China, X. Dong, F. Gallo, S. Gao, V. P. Ghate, S. Glienke, L. Goldberger, J. C. Hardin, C. Kuang, E. P. Luke, A. A. Matthews, M. A. Miller, R. Moffet, M. Pekour, B. Schmid, A. J. Sedlacek, R. A. Shaw, J. E. Shilling, A. Sullivan, K. Suski, D. P. Veghte, R. Weber, M. Wyant, J. Yeom, M. Zawadowicz, Z. Zhang, Aerosol and cloud experiments in the Eastern North Atlantic (ACE-ENA). *Bull. Am. Meteorol. Soc.* **103**, E619-E641 (2022).

69. G. Zheng, A. J. Sedlacek, A. C. Aiken, Y. Feng, T. B. Watson, S. Raveh-Rubin, J. Uin, E. R. Lewis, J. Wang, Long-range transported North American wildfire aerosols observed in marine boundary layer of eastern North Atlantic. *Environ. Int.* **139**, 105680 (2020).

70. S. L. von der Weiden, F. Drewnick, S. Borrmann, Particle Loss Calculator - A new software tool for the assessment of the performance of aerosol inlet systems. *Atmos. Meas. Tech.* **2**, 479–494 (2009).

71. L. Tigges, A. Wiedensohler, K. Weinhold, J. Gandhi, H. J. Schmid, Bipolar charge distribution of a soft X-ray diffusion charger. *J. Aerosol Sci.* **90**, 77–86 (2015).

72. M. D. Petters, S. M. Kreidenweis, A single parameter representation of hygroscopic growth and cloud condensation nucleus activity. *Atmos. Chem. Phys.* **7**, 1961–1971 (2007).

73. X. Gong, J. Zhang, B. Croft, X. Yang, M. M. Frey, N. Bergner, R. Y. W. Chang, J. M. Creamean, C. Kuang, R. V. Martin, A. Ranjithkumar, A. J. Sedlacek, J. Uin, S. Willmes, M. A. Zawadowicz, J. R. Pierce, M. D. Shupe, J. Schmale, J. Wang, Arctic warming by abundant fine sea salt aerosols from blowing snow. *Nat. Geosci.* **16**, 768–774 (2023).

74. R. Gelaro, W. McCarty, M. J. Suarez, R. Todling, A. Molod, L. Takacs, C. Randles, A. Darmenov, M. G. Bosilovich, R. Reichle, K. Wargan, L. Coy, R. Cullather, C. Draper, S. Akella, V. Buchard, A. Conaty, A. da Silva, W. Gu, G. K. Kim, R. Koster, R. Lucchesi, D. Merkova, J. E. Nielsen, G. Partyka, S. Pawson, W. Putman, M. Rienecker, S. D. Schubert, M. Sienkiewicz, B. Zhao, The Modern-Era Retrospective Analysis for Research and Applications, version 2 (MERRA-2). *J. Climate* **30**, 5419–5454 (2017).

75. H. Hersbach, B. Bell, P. Berrisford, S. Hirahara, A. Horányi, J. Muñoz-Sabater, J. Nicolas, C. Peubey, R. Radu, D. Schepers, A. Simmons, C. Soci, S. Abdalla, X. Abellan, G. Balsamo, P. Bechtold, G. Biavati, J. Bidlot, M. Bonavita, G. De Chiara, P. Dahlgren, D. Dee, M. Diamantakis, R. Dragani, J. Flemming, R. Forbes, M. Fuentes, A. Geer, L. Haimberger, S. Healy, R. J. Hogan, E. Hólm, M. Janisková, S. Keeley, P. Laloyaux, P. Lopez, C. Lupu, G. Radnoti, P. de Rosnay, I. Rozum, F. Vamborg, S. Villaume, J. N. Thépaut, The ERA5 global reanalysis. *Q. J. Roy. Meteorol. Soc.* **146**, 1999–2049 (2020).

76. H. Günther, S. Hasselmann, P. A. Janssen, "The WAM model cycle 4 (revised version)" (Deutsches Klimarechenzentrum, 1992).

77. J.-R. Bidlot, in *Workshop on Ocean Waves* (European Centre for Medium-Range Weather Forecasts, 2012), pp. 25–27.

78. J. Wang, Y. Wang, Evaluation of the ERA5 significant wave height against NDBC buoy data from 1979 to 2019. *Mar. Geod.* **45**, 151–165 (2021).

79. T. H. Bertram, R. E. Cochran, V. H. Grassian, E. A. Stone, Sea spray aerosol chemical composition: Elemental and molecular mimics for laboratory studies of heterogeneous and multiphase reactions. *Chem. Soc. Rev.* **47**, 2374–2400 (2018).

80. P. K. Quinn, T. S. Bates, K. S. Schulz, D. J. Coffman, A. A. Frossard, L. M. Russell, W. C. Keene, D. J. Kieber, Contribution of sea surface carbon pool to organic matter enrichment in sea spray aerosol. *Nat. Geosci.* **7**, 228–232 (2014).

81. D. B. Collins, T. H. Bertram, C. M. Sultana, C. Lee, J. L. Axson, K. A. Prather, Phytoplankton blooms weakly influence the cloud forming ability of sea spray aerosol. *Geophys. Res. Lett.* **43**, 9975–9983 (2016).

82. M. Pitchford, W. Maim, B. Schichtel, N. Kumar, D. Lowenthal, J. Hand, Revised algorithm for estimating light extinction from IMPROVE particle speciation data. *J. Air Waste Manage. Assoc.* **57**, 1326–1336 (2007).

83. F. Gallo, J. Uin, S. Springston, J. Wang, G. Zheng, C. Kuang, R. Wood, E. B. Azevedo, A. McComiskey, F. Mei, A. Theisen, J. Kyrouac, A. C. Aiken, Identifying a regional aerosol baseline in the eastern North Atlantic using collocated measurements and a mathematical algorithm to mask high-submicron-number-concentration aerosol events. *Atmos. Chem. Phys.* **20**, 7553–7573 (2020).

84. M. D. Anguelova, M. H. Bettenhausen, Whitecap fraction from satellite measurements: Algorithm description. *J. Geophys. Res. Oceans* **124**, 1827–1857 (2019).

85. S. J. Norris, I. M. Brooks, D. J. Salisbury, A wave roughness Reynolds number parameterization of the sea spray source flux. *Geophys. Res. Lett.* **40**, 4415–4419 (2013).

86. M. H. Sharqawy, J. H. Lienhard, S. M. Zubair, Thermophysical properties of seawater: A review of existing correlations and data. *Desalin. Water Treat.* **16**, 354–380 (2010).

87. E. W. Kolstad, T. J. Bracegirdle, I. A. Seierstad, Marine cold-air outbreaks in the North Atlantic: Temporal distribution and associations with large-scale atmospheric circulation. *Climate Dynam.* **33**, 187–197 (2008).

88. F. Ardhuin, Y. Quilfen, J. A. Hanafin, J. Sienkiewicz, P. Queffeulou, M. Obrebski, B. Chapron, N. Reul, F. Collard, D. Corman, E. B. de Azevedo, D. Vandemark, E. Stutzmann, Phenomenal

sea states and swell from a North Atlantic storm in February 2011: A comprehensive analysis. *Bull. Am. Meteorol. Soc.* **93**, 1825–1832 (2012).

89. A. F. Stein, R. R. Draxler, G. D. Rolph, B. J. B. Stunder, M. D. Cohen, F. Ngan, NOAA's HYSPLIT atmospheric transport and dispersion modeling system. *Bull. Am. Meteorol. Soc.* **96**, 2059–2077 (2015).

90. J. Wang, Y.-N. Lee, P. H. Daum, J. Jayne, M. Alexander, Effects of aerosol organics on cloud condensation nucleus (CCN) concentration and first indirect aerosol effect. *Atmos. Chem. Phys.* **8**, 6325–6339 (2008).

91. J. Liu, Z. Li, Aerosol properties and their influences on low warm clouds during the Two-Column Aerosol Project. *Atmos. Chem. Phys.* **19**, 9515–9529 (2019).

92. A. M. Aldhaif, D. H. Lopez, H. Dadashazar, D. Painemal, A. J. Peters, A. Sorooshian, An aerosol climatology and implications for clouds at a remote marine site: Case study over Bermuda. *J. Geophys. Res. Atmos.* **126**, e2020JD034038 (2021).

93. X. Gong, H. Wex, J. Voigtländer, K. W. Fomba, K. Weinhold, M. van Pinxteren, S. Henning, T. Müller, H. Herrmann, F. Stratmann, Characterization of aerosol particles at Cabo Verde close to sea level and at the cloud level – Part 1: Particle number size distribution, cloud condensation nuclei and their origins. *Atmos. Chem. Phys.* **20**, 1431–1449 (2020).

94. H. Wex, K. Dieckmann, G. C. Roberts, T. Conrath, M. A. Izaguirre, S. Hartmann, P. Herenz, M. Schäfer, F. Ditas, T. Schmeissner, S. Henning, B. Wehner, H. Siebert, F. Stratmann, Aerosol arriving on the Caribbean island of Barbados: Physical properties and origin. *Atmos. Chem. Phys.* **16**, 14107–14130 (2016).

95. S. Zhou, Y. Chen, A. Paytan, H. Li, F. Wang, Y. Zhu, T. Yang, Y. Zhang, R. Zhang, Non-marine sources contribute to aerosol methanesulfonate over coastal seas. *J. Geophys. Res. Atmos.* **126**, e2021JD034960 (2021).

96. C. Zhu, K. Kawamura, B. Kunwar, Organic tracers of primary biological aerosol particles at subtropical Okinawa Island in the western North Pacific Rim. *J. Geophys. Res. Atmos.* **120**, 5504–5523 (2015).

97. J. Li, G. Michalski, P. Davy, M. Harvey, T. Katzman, B. Wilkins, Investigating source contributions of size-aggregated aerosols collected in Southern Ocean and Baring Head, New Zealand using sulfur isotopes. *Geophys. Res. Lett.* **45**, 3717–3727 (2018).

98. K. Tsigaridis, D. Koch, S. Menon, Uncertainties and importance of sea spray composition on aerosol direct and indirect effects. *J. Geophys. Res. Atmos.* **118**, 220–235 (2013).
